# Hematopoietic progenitor kinase 1 inhibits the development and progression of pancreatic intraepithelial neoplasia

**DOI:** 10.1172/JCI163873

**Published:** 2023-06-15

**Authors:** Hua Wang, Rohan Moniruzzaman, Lei Li, Baoan Ji, Yi Liu, Xiangsheng Zuo, Reza Abbasgholizadeh, Jun Zhao, Guangchao Liu, Ruiqi Wang, Hongli Tang, Ryan Sun, Xiaoping Su, Tse-Hua Tan, Anirban Maitra, Huamin Wang

**Affiliations:** 1 Department of Gastrointestinal Medical Oncology and; 2 Department of Pathology, The University of Texas MD Anderson Cancer Center, Houston, Texas, USA.; 3 Department of Cancer Biology, Mayo Clinic, Jacksonville, Florida, USA.; 4 Advanced Technology Genomics Core,; 5 Department of Biostatistics, and; 6 Department of Bioinformatics & Computational Biology, The University of Texas MD Anderson Cancer Center, Houston, Texas, USA.; 7 Immunology Research Center, National Health Research Institutes, Zhunan, Taiwan.; 8 Department of Translational Molecular Pathology and; 9 Sheikh Ahmed Center for Pancreatic Cancer Research, The University of Texas MD Anderson Cancer Center, Houston, Texas, USA.

**Keywords:** Gastroenterology, Mouse models, Protein kinases, Tumor suppressors

## Abstract

Ras plays an essential role in the development of acinar-to-ductal metaplasia (ADM) and pancreatic ductal adenocarcinoma (PDAC). However, mutant *Kras* is an inefficient driver for PDAC development. The mechanisms of the switching from low Ras activity to high Ras activity that are required for development and progression of pancreatic intraepithelial neoplasias (PanINs) are unclear. In this study, we found that hematopoietic progenitor kinase 1 (HPK1) was upregulated during pancreatic injury and ADM. HPK1 interacted with the SH3 domain and phosphorylated Ras GTPase-activating protein (RasGAP) and upregulated RasGAP activity. Using transgenic mouse models of *HPK1* or *M46*, a kinase-dead mutant of *HPK1*, we showed that HPK1 inhibited Ras activity and its downstream signaling and regulated acinar cell plasticity. M46 promoted the development of ADM and PanINs. Expression of M46 in *Kras^G12D^ Bac* mice promoted the infiltration of myeloid-derived suppressor cells and macrophages, inhibited the infiltration of T cells, and accelerated the progression of PanINs to invasive and metastatic PDAC, while HPK1 attenuated mutant *Kras*–driven PanIN progression. Our results showed that HPK1 plays an important role in ADM and the progression of PanINs by regulating Ras signaling. Loss of HPK1 kinase activity promotes an immunosuppressive tumor microenvironment and accelerates the progression of PanINs to PDAC.

## Graphical abstract

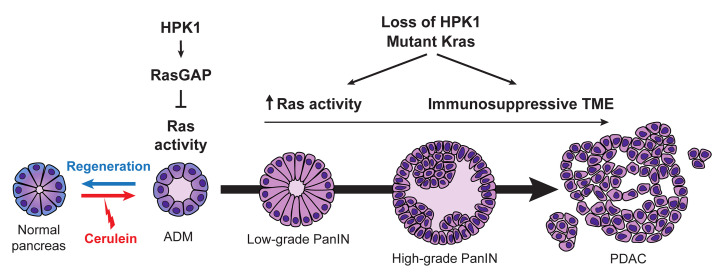

## Introduction

The high plasticity of pancreatic acinar cells allows them to transdifferentiate into a progenitor-like cell type with ductal traits, a process that is termed acinar-to-ductal metaplasia (ADM). ADM plays an essential role in pancreas regeneration after injury and may represent an adaptive response to tissue damage (1). Data from animal models show that ADM is mediated by the activation of Ras signaling and involves the rapid shutdown of pancreatic enzyme expression (1). In response to oncogenic signaling, the cells in ADM may evolve and develop pancreatic intraepithelial neoplasia (PanIN), which eventually progresses to pancreatic ductal adenocarcinoma (PDAC) (1). Since the vast majority (~80%) of patients with PDAC are diagnosed with locally advanced or metastatic disease, the prognosis for PDAC patients is poor (2, 3). Understanding the mechanisms involved in the initiation of pancreatic precursor lesions and their progression to invasive PDAC is important in order to discover potential molecular markers for early detection and possible targets for early intervention.

Oncogenic *Kras* mutations are present in more than 90% of human PDAC samples and play an important role in pancreatic tumorigenesis (4). Ras proteins function as membrane-bound GTPases that are stimulated by growth factor receptor tyrosine kinases and activate downstream effector pathways when bound to GTP. Several Ras effector pathways, including Raf/MEK/ERK (MAPK) and PI3K/Akt cascades, promote cell proliferation, differentiation, and survival (5). Ras activity is regulated by guanine nucleotide exchange factors (GEFs), which promote the transition from the inactive guanosine diphosphate–bound (GDP-bound) to the active GTP-bound conformation, and the Ras GTPase-activating proteins (RasGAPs). The intrinsic GTPase activity of Ras, which hydrolyzes the bound GTP into GDP, is inefficient for Ras inactivation. RasGAPs accelerate Ras inactivation by activating its GTPase activity (6).

Mutant *Kras* has low Ras activity and is an inefficient driver for the progression of early PanINs to PDAC in the genetically engineered mouse models (7–10). Additional molecular alterations or environmental stimuli, such as inflammation or tissue injury, are required to achieve high Ras activity, which drives the development of PanINs and their progression to PDAC (7–10). High Ras activity is also required for the maintenance of PDAC in the *iKras* (the doxycycline-inducible *Kras^G12D^*) model (11). It is important to point out that wild-type *Kras* and loss of heterozygosity of the *Kras* gene also play important roles in the growth of epithelial cells with mutant *Kras* and their progression to invasive carcinomas (12–15). However, the molecular mechanisms that regulate Ras activity during the initiation and progression of PanIN and the intrinsic differences in the susceptibility of pancreatic cells with different lineages to oncogenic mutant *Kras* remain unclear.

Hematopoietic progenitor kinase 1 (HPK1) is a serine/threonine kinase of the Ste20 family and functions as a MAP4K to activate MAP3Ks and the downstream MAP kinase pathway, one of the major Ras signaling pathways (16, 17). HPK1 kinase activity plays an important role in regulating T cell function mainly through the activation of NF-κB and JNK and inhibition of MEK1/2-mediated Erk activation (18, 19). Inhibiting HPK1 kinase function by knocking in the kinase-dead mutant of *HPK1*, *M46*, in which the ATP-binding lysine-46 residue is mutated to methionine, increases T cell receptor signaling, cytokine secretion, and CD8^+^ T cell function, and inhibits tumor growth in *M46*-transgenic mice (20, 21). Similarly, knockout of *HPK1* or inhibition of HPK1 kinase activity using small-molecular inhibitors has also been shown to enhance T cell antitumor functions and to inhibit tumor growth in syngeneic allograft mouse models (19, 22, 23). Clinical trials that target HPK1 kinase activity in immune cells for cancer immunotherapy are currently ongoing. Thus, a better understanding of how HPK1 impacts *Kras*-induced pancreatic precursor lesions and their progression to invasive PDAC is needed to develop precision therapeutics.

Recently, we and other groups showed that HPK1 is expressed in epithelial cells and carcinomas, including breast cancer, pancreatic cancer, and extramammary Paget’s disease (24–26). Our previous study showed that HPK1 is expressed in normal pancreatic ductal cells, but is lost in more than 90% of human PDAC samples, owing to proteasomal degradation mediated by the CUL7/Fbxw8 ubiquitin ligase (25, 27). Loss of HPK1 protein expression occurs in early PanINs and is strongly associated with progression from early PanIN to PDAC (25). Restoration of HPK1 expression in PDAC cells by proteasome inhibitors such as MG132 causes cell cycle arrest and growth inhibition, partly as a result of the stabilization of p21 and p27 (25). We also showed that HPK1 negatively regulates the oncogenic receptor tyrosine kinase Axl in PDAC cells through an endocytic pathway and decreases Axl downstream signaling and invasion (28, 29). Our studies suggest that HPK1 may function as a novel tumor suppressor in pancreatic cancer. Consistent with this notion, HPK1 expression correlates with better survival in estrogen receptor–positive breast cancer (26). Since both *Kras* mutation and loss of HPK1 occur in early PanINs, it is possible that HPK1 is involved in Ras signaling and *Kras*-driven initiation of early pancreatic precursor lesions and their progression to PDAC. To determine the functions of HPK1 during pancreatic tumorigenesis, we developed novel transgenic mouse models that target *HPK1* or its kinase-dead mutant, *M46*, in the pancreas using the elastase I promoter, referred to as *Bac-cre*, either alone or in mice with *Kras^G12D^*. Our study demonstrated that HPK1 kinase activity plays an important role in the development of ADM and PanINs. Wild-type HPK1 inhibits Ras activation through upregulation of RasGAP activity and inhibits *Kras^G12D^*-driven development of PanINs and their progression to invasive PDAC. Our results revealed not only the novel mechanisms by which the first reported serine/threonine kinase, HPK1, regulates Ras activity during early pancreatic tumorigenesis, but also the tumor suppressor role of HPK1 during the progression of pancreatic precursor lesions to PDAC.

## Results

### HPK1 expression is increased in ADM and low-grade PanINs, but is lost in high-grade PanINs and PDAC.

**Figure 1:**
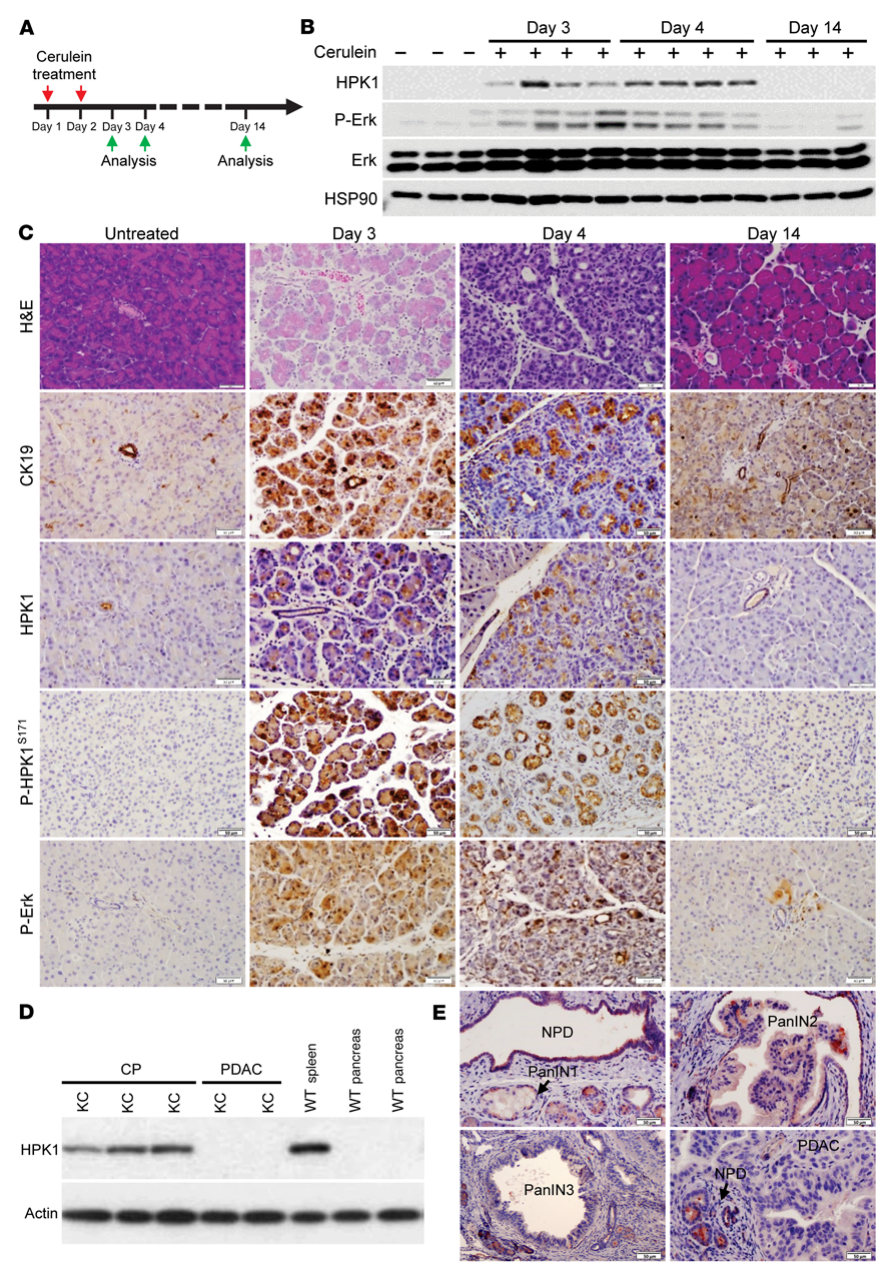
Expression of HPK1 in pancreas, ADM, PanINs, and PDAC. (**A**) Scheme of mouse treatment and tissue harvesting. Wild-type mice were treated with 8 hourly intraperitoneal cerulein injections (50 μg/kg; 3–4 mice in each group). (**B** and **C**) Expression of HPK1 and activation of HPK1 (p-HPK1^S171^) and Erk are increased in ADM at days 3 and 4, which is highlighted by positive staining for CK19; they return to normal at day 14. Scale bars: 50 µm. (**D**) Immunoblots show that HPK1 is expressed in chronic pancreatitis (CP) but lost in PDAC from KC mice. (**E**) HPK1 is expressed in PanIN1 but lost in high-grade PanINs and PDAC from KC mice. Positive staining in normal pancreatic ductal cells (NPD) served as internal positive controls. Scale bars: 50 μm.

To examine HPK1 expression during pancreatic injury, we treated wild-type mice with intraperitoneal injections of cerulein (ICer) for 2 days (Figure 1A). We observed significantly increased HPK1 expression in the pancreata at days 3 and 4 after the initiation of ICer, which was accompanied by increased Erk activation. Both HPK1 expression and activation of Erk returned to the levels of normal pancreata at day 14 by immunoblotting (Figure 1, A and B). Histologic analyses showed severe injury of the exocrine pancreas and extensive ADM, which was highlighted by positive CK19 staining, at days 3 and 4. The pancreas fully regenerated and was histologically normal at day 14 (Figure 1C). By immunohistochemistry (IHC), both HPK1 expression and Erk activation were increased in ADM at days 3 and 4 and reduced to those of normal pancreas at day 14. Expression of phosphorylated HPK1^S171^ (p-HPK1^S171^), which was one of the major HPK1 autophosphorylation sites and served as a marker for HPK1 activation and kinase activity, was also increased in ADM at days 3 and 4 and reduced to that of normal pancreas at day 14 (Figure 1C). To examine HPK1 expression in mutant *Kras*-driven PDAC models, we observed increased HPK1 expression in chronic pancreatitis tissue from *LSL-Kras^G12D^ Ela-CreERT* mice (KC) mice treated with ICer (Figure 1D). HPK1 was expressed in normal pancreatic ducts and low-grade PanINs, but not in high-grade PanINs or PDAC (Figure 1, D and E). Similar results were observed in PanINs and PDAC samples from *LSL-Kras^G12D^ Pdx-Cre* mice (Supplemental Figure 1; supplemental material available online with this article; https://doi.org/10.1172/JCI163873DS1). These data suggest that HPK1 is involved in pancreatic injury, development of ADM, and progression of low-grade PanIN to high-grade PanIN and PDAC in mutant *Kras*–driven PDAC models.

### Generation of transgenic mouse models for pancreas-specific expression of HPK1 or its kinase-dead mutant, M46.

To study the functions of *HPK1* in pancreatic tumorigenesis, we generated green fluorescent protein (*GFP*)*–HPK1* or *GFP*/*M46*-transgenic mice (Supplemental Figure 2, A and B) and crossed them with tamoxifen-inducible *Ela-CreERT* (hereafter referred to as *Bac-cre*) mice to express *HPK1* or *M46* in the pancreas (30). Consistent with the Cre activity, the pancreata of *HPK1 Bac-cre* (HC) and *M46 Bac-cre* (MC) mice lost GFP fluorescence and expressed HPK1 or M46 in acinar cells (Supplemental Figure 2, C–H). As expected, the pancreata of HC mice had high basal and cerulein-induced HPK1 kinase activities and p-HPK1^S171^ expression while minimal kinase activity was detected in both untreated and treated MC mice (Supplemental Figure 2I). Both HC and MC mice were born with the expected Mendelian frequencies and body weights similar to those of wild-type controls. Their organs appeared to be normally formed. Their pancreata showed normal size, parenchymal architecture, histology, and cytodifferentiation up to the age of 18 months, the longest time examined (*n* = 10). There were no differences in survival between HC or MC mice and control mice (data not shown). Thus, acinar cell–specific expression of HPK1 or M46 did not result in any major abnormalities in the pancreas.

### HPK1 inhibits Ras signaling through RasGAP, inflammatory pathways, and epithelial reprogramming.

**Figure 2:**
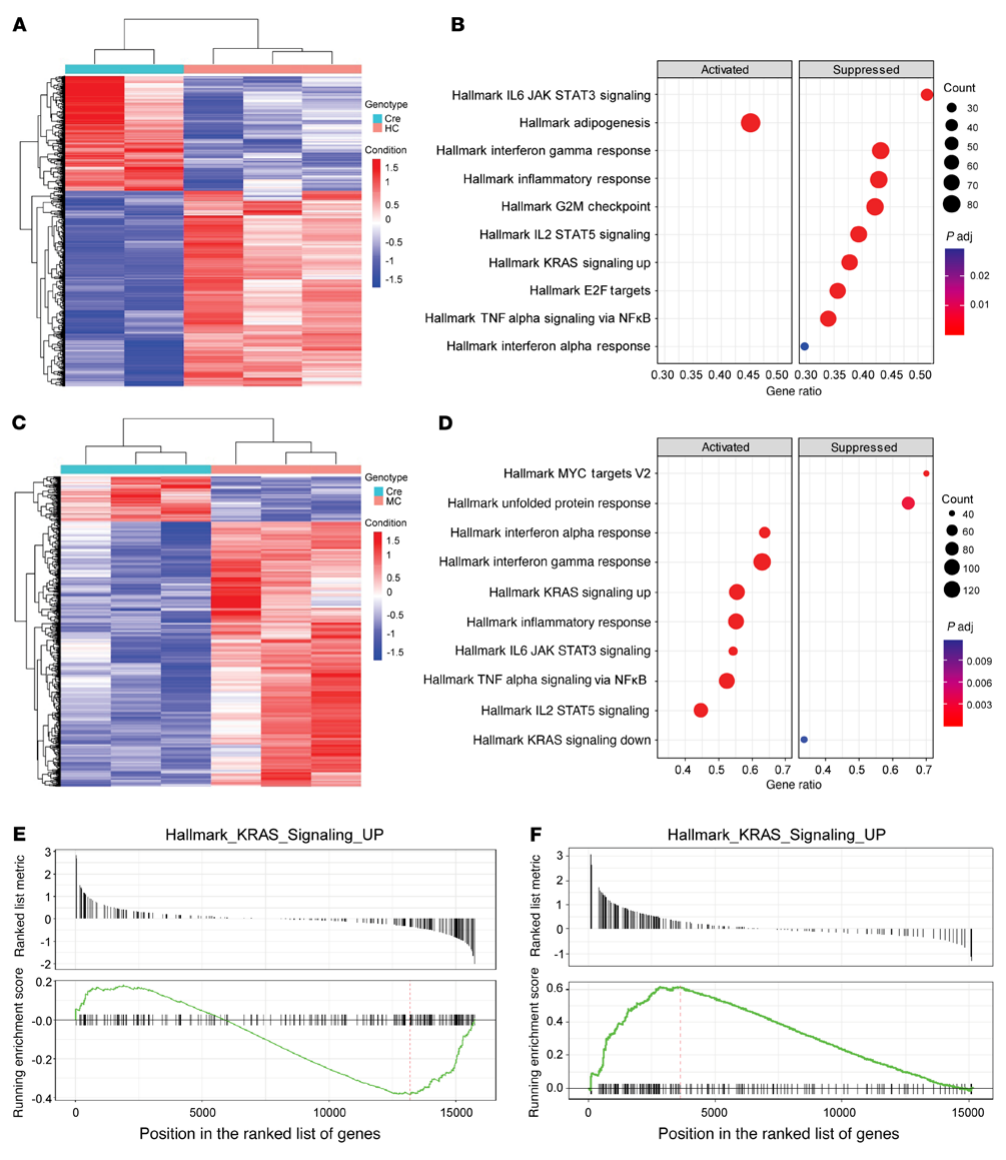
HPK1 regulates Ras signaling and inflammatory pathways. (**A** and **C**) Heatmaps of the differentially expressed genes by RNA-Seq in the pancreata of HC and MC mice treated with cerulein (*n* = 6). (**B** and **D**) Dot plots of the top 10 activated and suppressed hallmark pathways in HC and MC mice by gene set enrichment analysis. (**E** and **F**) Gene set enrichment analyses demonstrated that the Kras signaling upregulated gene set was suppressed in the pancreata of HC mice (**E**), but activated in MC mice (**F**).

To investigate whether HPK1 was involved in acinar cell reprogramming and to identify the mechanisms that are responsible for ADM, HC, MC, and littermate control mice (*n* = 6 for each group, 6–8 weeks old) were treated with ICer to induce HPK1 kinase activity. The pancreata were harvested 24 hours after ICer, and comparative RNA-Seq transcriptome analyses were performed. The RNA-Seq yielded around 20 million read pairs per sample on average, and about 80% of reads were uniquely aligned to the mouse genome. The results of RNA-Seq analyses for HC and MC mice are shown in Figure 2. Gene set enrichment analyses of the differentially expressed genes (cutoff criteria: adjusted *P* value < 0.05) using hallmark gene sets showed that Kras signaling, IFN-α and -γ response, inflammatory response, IL-6/JAK/STAT3 signaling, IL-2/STAT5 signaling, and TNF-α signaling via NF-κB were among the top 10 enriched pathways in both HC and MC mice. Expression of HPK1 inhibited these pathways in HC mice, while expression of M46 led to the activation of these pathways in MC mice (Figure 2, B and D–F). The cnetplot displayed a network of differentially expressed genes strongly linking M46 expression with inflammatory and Kras signaling pathways (Supplemental Figure 3).

**Figure 3:**
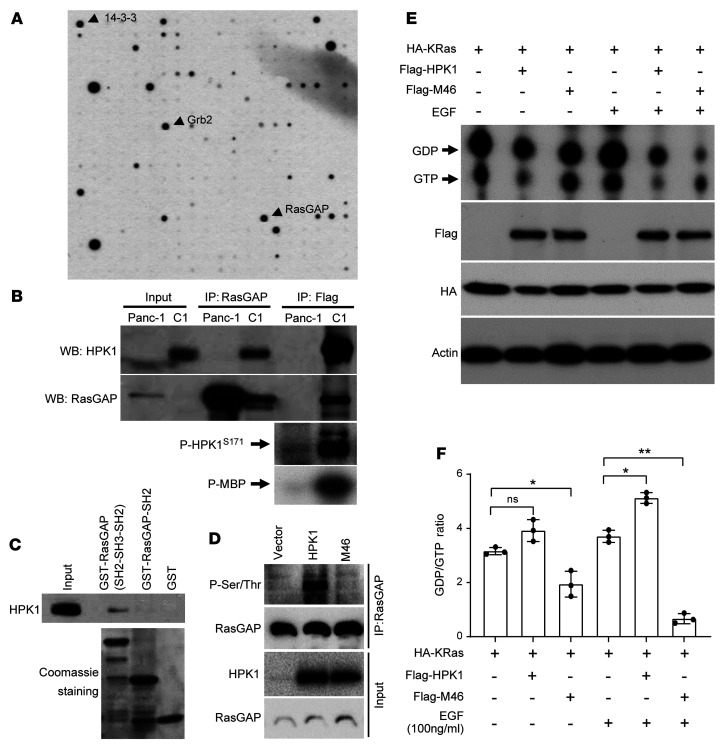
HPK1 regulates Ras signaling pathways through RasGAP. (**A**) HPK1-interacting proteins identified using an antibody-based array and cell lysates from Panc-1/*HPK1* stable cells treated with 2.0 μM MG132 to stabilize HPK1 protein. Immunodetection was performed using anti-FLAG antibody. (**B**) HPK1 interacts with RasGAP in Panc-1/*HPK1* stable cells (C1) treated with 2.0 μM MG132. HPK1 kinase activity and autophosphorylation (p-HPK1^S171^) are shown in the lower two panels. (**C**) GST pull-down assays show that RasGAP SH2-SH3-SH2 domains can effectively pull down HPK1 protein. No HPK1 was pulled down using RasGAP SH2 domain or GST alone. (**D**) RasGAP is phosphorylated at serine/threonine residue(s) by HPK1 but not M46. *HPK1* or *M46* was transfected into HEK293T cells. RasGAP was immunoprecipitated and probed with phospho–serine/threonine antibody. (**E** and **F**) In vitro RasGAP activity assays show that HPK1 enhances both basal and EGF-stimulated RasGAP activity as reflected by increased GDP/GTP ratio while M46 inhibits both basal and EGF-stimulated RasGAP activity. FLAG-HPK1 or FLAG-M46 was cotransfected with HA-Ras into HEK293T cells. Transfected cells were either untreated or treated with 100 ng/mL EGF for 10 minutes. The effect of HPK1 on RasGAP activity was determined by measurement of GTP- and GDP-bound Ras using thin-layer chromatography (**E**). (**F**) GTP- and GDP-bound Ras was quantified using ImageJ (NIH), and GDP/GTP ratios were plotted. Experiments were repeated 3 times. Data are presented as mean ± SEM. Statistical significance was determined by paired-samples *t* test using the cells transfected with *Kras* alone either untreated or treated with EGF as controls. When untreated, cotransfection of *M46* with *Kras* significantly reduced the GDP/GTP ratio. Cotransfection of HPK1 with *Kras* increased the GDP/GTP ratio, but the *P* value was not significant. When treated with EGF, cotransfection of *HPK1* with *Kras* significantly increased the GDP/GTP ratio, while cotransfection of *M46* with *Kras* significantly reduced the GDP/GTP ratio. *P < 0.05; **P < 0.01.

To identify HPK1-interacting proteins, we performed an antibody-based array study using *Panc-1*/*HPK1* stable cells, which were treated with 2.0 μM MG132 to stabilize HPK1 protein, and identified approximately 20 candidate HPK1-interacting proteins. One of the identified HPK-interacting proteins was RasGAP, which binds RasGTP, promotes GTP hydrolysis, and attenuates Ras activity and its downstream signaling. We also identified other proteins that play an important role in the Ras pathway, including Grb2 and 14-3-3 (Figure 3A), both of which have been shown previously to interact with HPK1 in TCR signaling (31, 32) and thus served as internal markers that validated our screening for HPK1-interacting proteins. HPK1 interacted with RasGAP in Panc-1/*HPK1* stable cells by coimmunoprecipitation (Figure 3B). To map the RasGAP domains that are involved in its interaction with HPK1, we performed a GST pull-down assay using different GST fusion proteins. We showed that the SH2-SH3-SH2 domain (171 to 448) of RasGAP can effectively pull down HPK1 protein. No HPK1 protein was pulled down using the SH2 domain (175 to 274) or GST alone (Figure 3C). These data suggest that RasGAP interacts with HPK1 mainly through its SH3 domain.

To examine whether HPK1 could phosphorylate RasGAP, *HPK1* or *M46* was transfected into HEK293T cells. Endogenous RasGAP was immunoprecipitated and probed with phospho–serine/threonine antibody. RasGAP was phosphorylated at serine/threonine residue(s) by HPK1, but not M46 (Figure 3D). To study the functions of HPK1 in regulating RasGAP, we performed RasGAP activity assays as previously described (33). HPK1 or M46 was cotransfected with Ras into HEK293T cells. The transfected cells were either treated with EGF for 10 minutes or left untreated as control. We found that HPK1 enhanced both basal and EGF-stimulated RasGAP activity as reflected by the increased GDP/GTP ratio. In contrast, M46 inhibited both basal and EGF-stimulated RasGAP activity. When treated with EGF, cells cotransfected with M46 and Ras had a RasGAP activity 7.0 times lower than those transfected with Ras alone (Figure 3, E and F). These results suggest that HPK1 kinase activity is important in the phosphorylation and regulation of RasGAP activity and Ras signaling.

### HPK1 interacts with endogenous RasGAP and inhibits Ras activity and Ras signaling.

**Figure 4:**
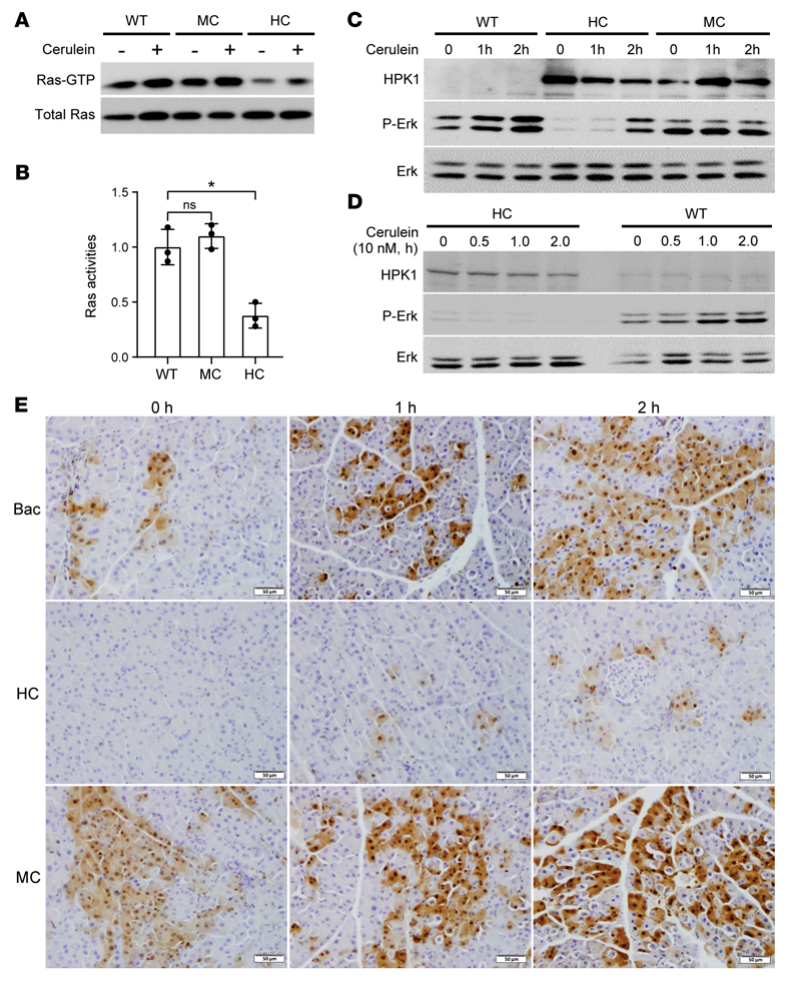
HPK1 inhibits Ras activities and Erk activation. *Bac-cre* control, HC, and MC mice were either untreated or treated with 1 dose of ICer to activate HPK1 and Ras pathways (*n* = 6 per group). The pancreata were harvested at 1 or 2 hours after the treatment. (**A**) Ras activity was measured by Raf-RBD pull-down assays. (**B**) Ras-GTP levels normalized with total Ras in MC and HC mice compared with wild-type mice after cerulein treatment. Data are presented as mean ± SEM; *P < 0.05. Unpaired *t* test was used to calculate *P* values. (**C** and **D**) HPK1 inhibits Erk activation in the pancreata (**C**) and primary cultures of acinar cells from HC mice treated with cerulein as indicated (**D**). (**E**) IHC analysis shows that the pancreata of HC mice have decreased activation of Erk. Scale bars: 50 μm.

To examine the functions of HPK1 in regulating Ras signaling in vivo, we first performed coimmunoprecipitation experiments and confirmed that HPK1 interacted with endogenous RasGAP in the pancreata of both HC and MC mice (Supplemental Figure 4). Expression of HPK1 inhibited Ras activity and Erk activation in the pancreata of HC mice compared with littermate control or MC mice (Figure 4, A–C). To rule out the possibility of mouse variation at different time points, we isolated the acinar cells from 3 HC and 3 control mice, split them into 4 identical dishes, treated them with cerulein, and harvested them at different time points as indicated. Erk activation was markedly reduced in acinar cells from HC mice compared with those from control mice (Figure 4D). The pancreata of HC mice had lower Erk activation than those of the MC and control mice (Figure 4E). These results suggest that HPK1 kinase activity was required to inhibit Ras activation and its downstream Erk signaling.

### Acinar cell–specific expression of M46 or HPK1 knockout promotes pancreatic inflammation, ADM, and PanIN formation.

**Figure 5:**
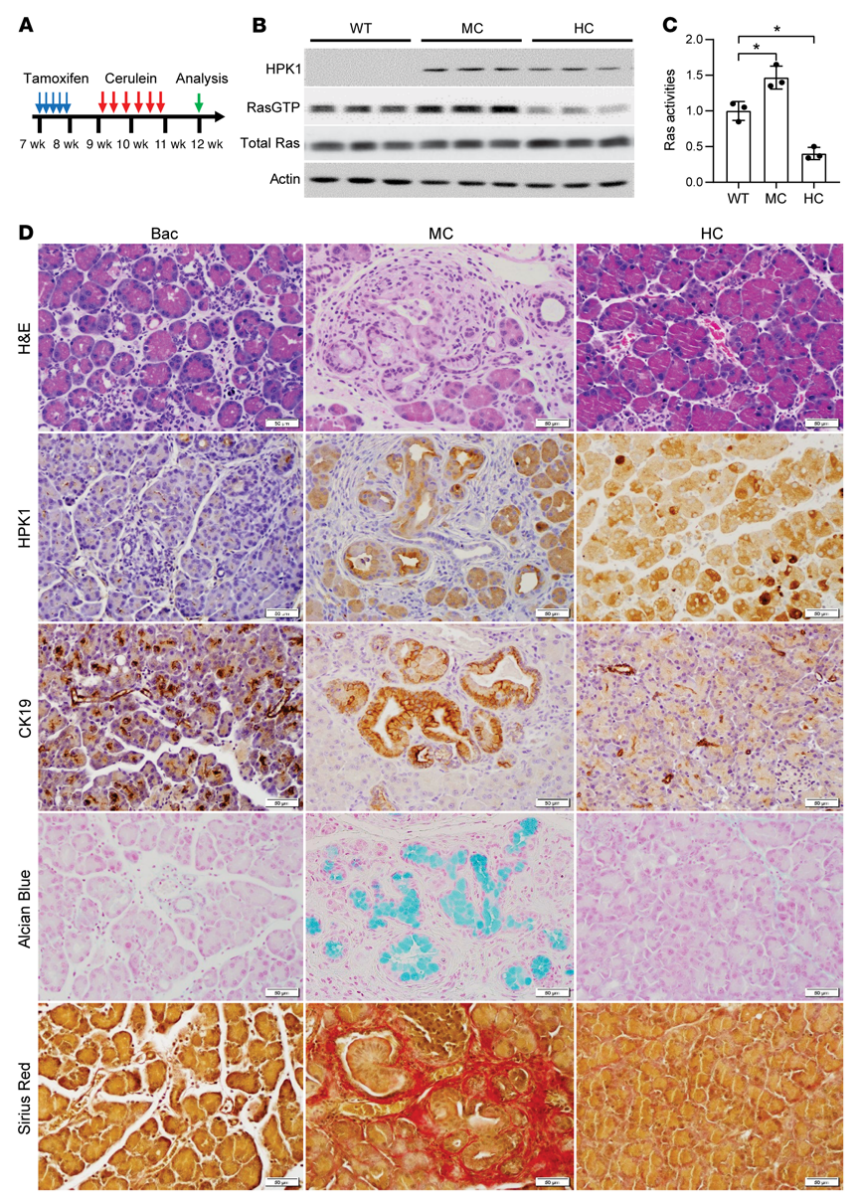
Acinar cell–specific expression of kinase-dead mutant M46 promotes pancreatic inflammation, ADM, and PanIN formation. (**A**) Scheme of cerulein treatment and tissue harvesting. (**B** and **C**) Acinar cell–specific expression of HPK1 inhibits Ras activity, while M46 increases Ras activity. (**C**) Ras activity was quantified using ImageJ and plotted. Data are presented as mean ± SEM; *P < 0.05. Unpaired *t* test was used to calculate *P* values. (**D**) Acinar cell–specific expression of M46 promotes the development of chronic pancreatitis, ADM, and PanINs. Shown is representative histology of pancreata from Bac control, MC, and HC mice by H&E; IHC for HPK1 and CK19; Alcian blue; and sirius red. PanINs are highlighted by positive staining for CK19 and Alcian blue. Sirius red highlights pancreatic fibrosis in treated MC mice. Scale bars: 50 μm.

To examine the function of HPK1 in pancreatic tumorigenesis, the HC, MC, and littermate control mice were treated with tamoxifen to induce acinar cell–specific expression of HPK1 or M46 followed by ICer treatment for 2 weeks (Figure 5A). Compared with the controls, MC mice had increased Ras activity while HC mice had decreased Ras activity (Figure 5, B and C). Expression of M46 promoted the development of chronic pancreatitis, ADM, and PanIN in 8/8 (100%), 8/8 (100%), and 5/8 (62.5%) mice, respectively. Similar results were observed in HPK1-knockout mice: chronic pancreatitis, ADM, and PanIN were observed in 10/10 (100%), 10/10 (100%), and 7/10 (70%) HPK1-knockout mice, respectively (Supplemental Figure 5). In contrast, all control (10/10) and HC mice (10/10) had minimal histologic changes in their pancreata with minimal ADM, and none of them developed PanINs (Figure 5D). We did not observe significant differences in histology between HC and control mice at day 20 after the initiation of ICer treatment. These results suggest that inhibition of HPK1 kinase activity by expression of M46 plays a critical role in Ras activation and the development of ADM and PanINs.

### Targeted expression of M46 promotes the development and progression of PanINs in Kras^G12D^ Bac-cre mice.

**Figure 6:**
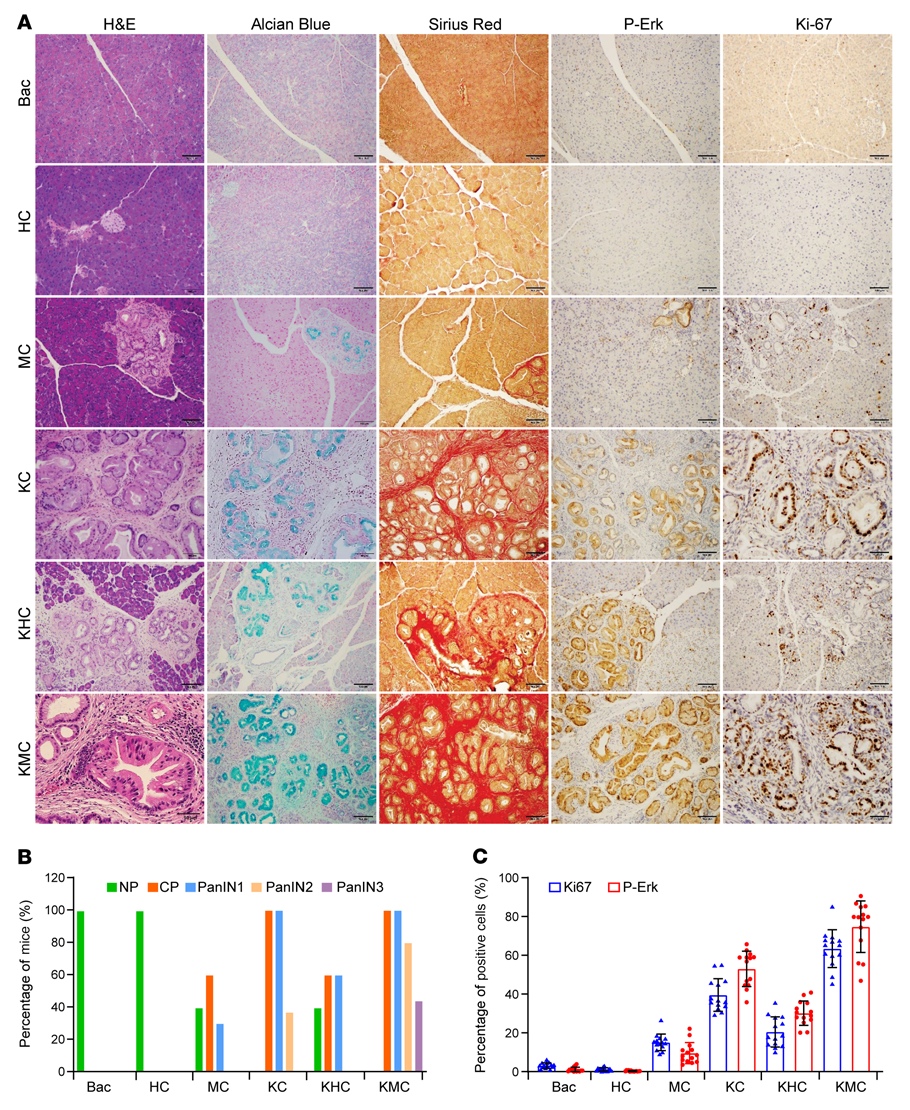
Kinase-dead mutant M46 promotes chronic pancreatitis, Erk activation, and progression of PanINs while HPK1 inhibits chronic pancreatitis and PanIN progression in KC mice. (**A**) Representative micrographs show histopathology, Alcian blue, sirius red, and IHC stains for p-Erk and Ki-67 of the pancreata from different groups. All mice were treated with tamoxifen, then with 8 doses of ICer for 2 days to induce pancreatitis. The pancreata were harvested at 28 days after completion of ICer treatment. Scale bars: 50 μm. (**B**) Bar graph shows the frequencies of normal pancreas (NP), chronic pancreatitis (CP), and different grades of PanINs from different groups (*n* = 20 per group). (**C**) Bar graph shows the average percentage of p-Erk–positive cells and Ki-67 proliferation index in pancreata from different groups. Data are presented as mean ± SEM. Unpaired *t* test was used to calculate *P* values.

**Figure 7:**
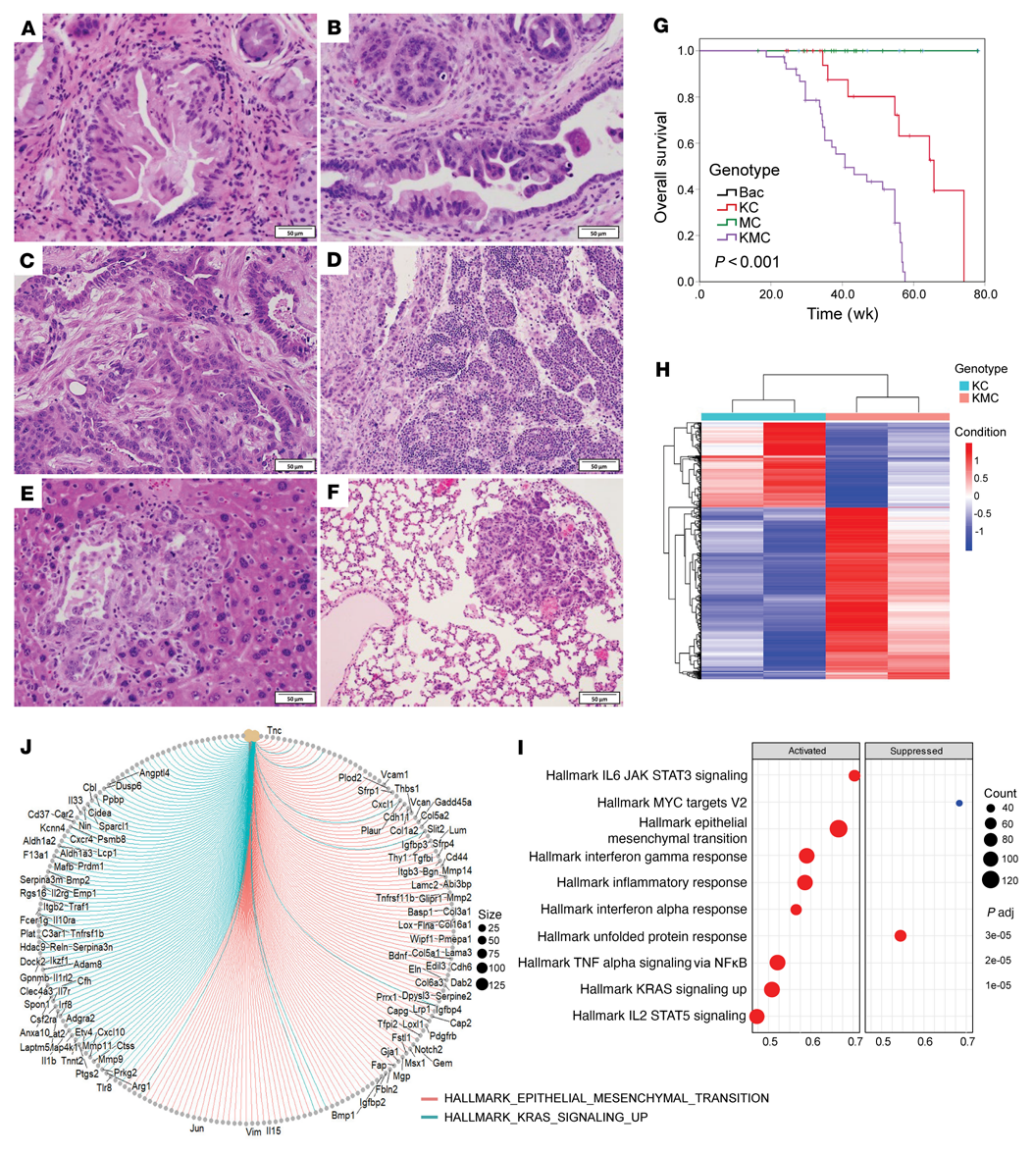
Acinar cell–specific expression of M46 promotes progression of PanINs to invasive and metastatic PDAC and decreases survival in KMC mice. (**A**–**F**) Representative micrographs show PanIN2 (**A**), PanIN3 (**B**), invasive PDAC (**C**), and metastatic PDAC in lymph node (**D**), liver (**E**), and lung (**F**) in KMC mice. Scale bars: 50 μm. (**G**) Kaplan-Meier survival curves show that KMC mice (*n* = 39) had shorter survival than KC (*n* = 29, *P* < 0.0001), MC (*n* = 34, *P* < 0.0001), or control mice (*n* = 9, *P* < 0.0001). The log-rank test was used to determine statistical significance. (**H**) Heatmaps of the differentially expressed genes in PDAC samples from KMC and KC mice (*n* = 6) by RNA-Seq. (**I**) Dot plot of the top 10 activated and suppressed hallmark pathways in PDAC samples of KMC mice by gene set enrichment analysis. (**J**) Gene set enrichment analyses demonstrated that the Kras signaling upregulated gene set and the epithelial-mesenchymal transition gene set were enriched in PDAC samples of KMC mice.

To examine the functions of HPK1 in *Kras^G12D^*-mediated formation of PanINs and their progression to invasive PDAC, *Kras^G12D^ Bac-cre* (KC), *Kras^G12D^ HPK1 Bac-cre* (KHC), and *Kras^G12D^ M46 Bac-cre* (KMC) mice were treated with tamoxifen followed by ICer treatment — which accelerate the development and progression of PanINs — for 2 days to induce pancreatitis. The pancreata were harvested and analyzed at 28 days after completion of cerulein treatment. KMC mice developed severe chronic pancreatitis (100%, 10/10), PanIN1 (100%, 10/10), PanIN2 (80%, 8/10), and PanIN3 (50%, 5/10). In comparison, PanIN2 was identified in only 25% of KC mice, but none of the KHC mice (0%, 0/10). None of the KC and KHC mice developed PanIN3 (0%, 0/10). KHC mice also had a significantly reduced amount of fibrosis and lower incidence of PanINs than KMC or KC mice (Figure 6, A and B). KMC mice had higher Erk activation and Ki-67 proliferation index than KC mice. In contrast, KHC mice had decreased Erk activation and lower Ki-67 labeling index than KC mice (Figure 6, A and C). To further examine the function of HPK1 kinase activity in PanIN progression and survival, we performed aging experiments on KC, KMC, KHC, MC, HC, and control mice for 18 months. During aging experiments, we observed a significant decrease in HPK1 kinase activity in KHC and HC mice 2–3 months after tamoxifen treatment, which could be due to the lack of stimulation, even though HPK1 protein was detectable. Therefore, the KHC and HC mice were not included in this aging experiment. KMC mice developed high-grade PanINs and PDAC starting at 6 months and metastatic PDAC to lymph node, liver, and lung as early as 7.5 months of age (Figure 7, A–F). In contrast, no KC mice developed PDAC at age 9–12 months. KMC mice had significantly shorter survival than KC, MC, or control mice (Figure 7G). The PDACs from KMC mice had lower apoptosis compared with those from KC mice (Supplemental Figure 6). RNA-Seq analyses of the PDAC samples from KMC mice at 9 months and KC mice at 15 months showed that expression of M46 was associated with upregulation of epithelial-mesenchymal transition in addition to upregulation of Kras signaling, inflammatory, and other hallmark pathways observed in pancreata of MC mice treated with cerulein (Figure 7, H–J). These data provided additional evidence that HPK1 inhibits Ras signaling and inflammatory pathways. Therefore, HPK1 kinase activity is required to inhibit ductal cell proliferation and the development and progression of PanINs in KC mice. Inhibition of HPK1 kinase activity by expression of kinase-dead M46 enhances mutant *Kras*–driven cell proliferation and accelerates PanIN progression to invasive and metastatic PDAC.

**Figure 8:**
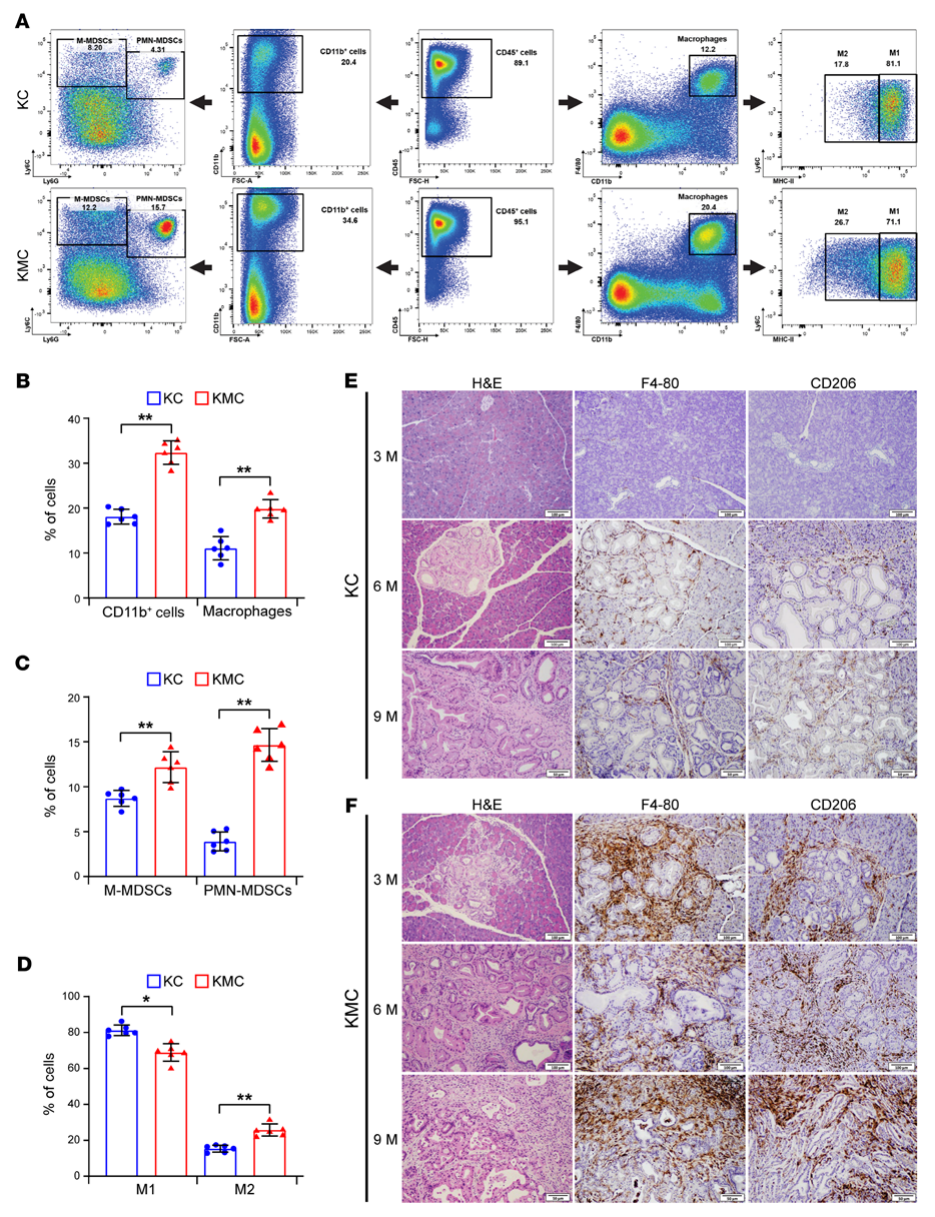
Kinase-dead mutant M46 promotes infiltration of MDSCs and macrophages in pancreas of KMC mice. (**A**–**D**) KC and KMC mice at 6 months were euthanized (*n* = 6 per group). The infiltrating MDSCs (CD11b^+^Ly6C^hi^Ly6G^–^) and macrophages in pancreas were quantified by flow cytometry. (**A**) Representative flow cytometry gating results for CD45^+^ cells, including CD11b^+^ cells, monocytic (M) MDSCs (CD11b^+^Ly6C^hi^Ly6G^–^), polymorphonuclear (PMN) MDSCs (CD11b^+^Ly6C^+^Ly6G^+^), macrophages (CD45^+^CD11b^+^F4/80^+^), Ly6C^lo^ macrophages with low MHC-II expression (M2), and Ly6C^lo^ macrophages with high MHC-II expression (M1), in KC and KMC mice. (**B** and **C**) Quantitative results for different CD45^+^ cell types, including CD11b^+^ cells and CD11b^+^F4/80^+^ cells (**B**) and M-MDSCs and PMN-MDSCs (**C**). (**D**) Quantitative results for M2 macrophages (CD11b^+^F4/80^+^Ly6C^lo^MHC-II^lo^) and M1 macrophages (CD11b^+^F4/80^+^Ly6C^lo^MHC-II^hi^). Data are presented as mean ± SEM. Unpaired *t* test was used to calculate *P* values. *P < 0.05; **P < 0.01. (**E** and **F**) Representative micrographs show pancreatic histology and IHC staining results for F4/80^+^ and CD206^+^ macrophages in KC and KMC mice at 3, 6, and 9 months (M). Scale bars: 100 μm for 3 months and 6 months; 50 μm for 9 months.

To examine the mechanisms by which M46 promoted pancreatic tumorigenesis, we examined the pancreatic immune microenvironment of the KMC and KC mice at age 6 months by flow cytometric analysis. We observed a significant increase in myeloid cells (CD11b^+^), including myeloid-derived suppressor cells (MDSCs; CD11b^+^Ly6C^hi^Ly6G^–^) and macrophages (CD45^+^CD11b^+^F4/80^+^), in KMC mice compared with sex- and age-matched KC littermates. Interestingly, the KMC mice had higher numbers of CD45^+^CD11b^+^F4/80^+^Ly6C^lo^MHC-II^lo^ macrophages, an indicator of their polarization into M2-like macrophages, than KC mice (Figure 8). In addition, KMC mice also had a lower number of CD3^+^CD8^+^ and CD3^+^CD4^+^ T cells and decreased CD4^+^IFN-γ^+^ and CD8^+^IFN-γ^+^ T cells than KC mice (Supplemental Figures 7 and 8). No significant difference in the infiltrating B cells was noted (Supplemental Figure 9). KMC mice had markedly increased F4/80^+^ and CD206^+^ macrophages compared with sex- and age-matched KC littermates at 3, 6, and 9 months (Figure 8, E and F). Our results suggested that expression of M46 in KC mice increases the recruitment of MDSCs and M2 macrophages into the pancreas, which orchestrates an immunosuppressive microenvironment and promotes the progression of PanINs to PDAC.

## Discussion

Many studies have demonstrated that oncogenic mutant *Kras* plays a key role in the development of PanINs and PDAC (34–43). However, mutant Kras alone has low Ras activity and is not an efficient driver for the development and progression of PanINs to invasive PDAC (7, 36). The orchestration between mutant Kras and additional genetic alterations or environmental stimuli is required to achieve high Ras activity, which is essential to accelerate the development and progression of PanINs (7, 36). Since both *Kras* mutation and loss of HPK1 protein expression occur during the early stages of pancreatic tumorigenesis, it is extremely important to examine the functions of HPK1 in regulating Ras activation and downstream signaling during the development and progression of ADM and PanINs. In this study, we demonstrated that pancreatic acinar cell–specific expression of *HPK1* kinase-dead mutant M46 promotes chronic pancreatitis, ADM, and PanIN formation without mutant *Kras*. We also demonstrated that M46 increases the recruitment of MDSCs and M2 macrophages, which orchestrates an immunosuppressive microenvironment, inhibits T cell infiltration and activation, and promotes the progression of PanINs to invasive and metastatic PDAC in KC mice. On the other hand, acinar cell–specific expression of HPK1 inhibits Ras activity and ADM development. More importantly, we demonstrated, for the first time to our knowledge, that HPK1 is a regulator of Ras signaling through RasGAP using both in vitro systems and our transgenic mouse models. Our findings provide new insights into the mechanisms of the regulation of Ras activity and the development and progression of ADM and PanINs.

Acinar cell plasticity in adult pancreas is involved in both pancreatic regeneration and pancreatic tumorigenesis. Previous studies have shown that cerulein-induced acute and chronic pancreatitis accelerates *Kras^G12D^*-mediated PanIN and PDAC formation (36, 44–48). In the absence of oncogenic *Kras*, acute pancreatitis induces transient ADM, which is followed by regeneration to normal pancreas (47, 49). In this study we demonstrated, for the first time to our knowledge, that HPK1 expression was increased in cerulein-induced acute pancreatitis and ADM, which synchronized with increased Ras activity and Erk activation. Acinar cell–specific expression of M46 alone is sufficient to inhibit pancreatic regeneration in cerulein-induced pancreatitis and to increase ADM and development of PanINs, which is attributed to increased Ras activity and its downstream signaling. Our data suggest that HPK1 kinase activity plays an essential role in acinar cell plasticity, regulation of Ras signaling, regeneration of pancreas, and the development of ADM and PanINs.

HPK1 has been shown to regulate NF-κB and JNK pathways and plays an important role in stress responses, proliferation, and apoptosis in hematopoietic cells. More importantly, HPK1 inhibits the MEK1/2-mediated Erk activation that is one of the major Ras effectors (18). However, the functions of HPK1 in *Ras*-mediated tumorigenesis are largely unknown. Only one previous study showed that shRNA knockdown of HPK1 in Wehi-231 lymphoma cells increased the activation of Ras-related protein 1 (Rap1) (50). Our study demonstrated that HPK1 interacts with RasGAP in human PDAC cells and the endogenous RasGAP in the pancreata of our new mouse models. HPK1 binds to RasGAP through its SH3 domain, phosphorylates RasGAP at putative serine/threonine residue(s), and increases RasGAP activity, which enhances the intrinsic Ras GTPase activity and downregulates oncogenic Ras and its downstream signaling. It has been shown that suppression of cellular RasGAP function leads to a decrease in intrinsic Ras GTPase activity and enhances Ras activation and cancer development (51–53). This HPK1/RasGAP pathway inhibiting Kras signaling may be essential for normal cellular functions and could partly explain why expression of mutant *Kras* at physiological levels in KC mice had little oncogenic effect in this study and previous studies (7, 54, 55). Our findings that HPK1 is upregulated during ADM, the earliest stage of pancreatic tumorigenesis, which synchronizes with and inhibits Ras activation and its downstream Erk activation, suggested a novel negative regulatory function of HPK1 in Ras signaling and *Kras*-driven pancreatic tumorigenesis. Loss of HPK1 or expression of kinase-dead mutant M46 would release the check on oncogenic Ras signaling and lead to dramatically amplified pathological effects and progression of PanINs to invasive PDAC. Consistent with this notion, we demonstrated that M46 promoted the *Kras^G12D^*-mediated development of PanINs and their progression to invasive and metastatic PDAC, while HPK1 inhibited the progression of PanINs in KC mice. Although RasGAP is considered inactive toward mutant Ras, a recent study showed that NF1 RasGAP stimulates GTP hydrolysis when bound to *Kras^G13D^* (53).

The results from our RNA-Seq analyses of the pancreata of KC and MC mice showed that expression of M46 was associated with activation of Kras signaling and several hallmark pathways involved in inflammatory responses while expression of HPK1 had the opposite effects. After ICer treatment, we observed similar frequencies of chronic pancreatitis, ADM, or PanIN formation in MC mice compared with HPK1-knockout mice. Although our Cre-inducible transgenic mice had endogenous HPK1 expression, our data were consistent with a recent study by Hernandez et al., which carefully compared the function of HPK1-knockout mice and M46-knockin mice and found that M46 functions similarly to HPK1 knockout in Erk activation and regulation of CD8^+^ T cell responses (20). These data do not support the gain of function of M46, although we cannot exclude the possibility that M46 may have additional gain of function.

The kinase activity of HPK1 plays an important role in regulating T cell functions (18, 19). Recent studies demonstrated that inhibition of HPK1 kinase function by knockout of HPK1, knockin of kinase-dead mutant M46, or inhibition of HPK1 kinase activity using small-molecular inhibitors enhances the antitumor T cell functions and inhibition of tumor growth in synergistic allograft mouse models (19–23). In this study, we showed that targeted expression of M46 in pancreatic acinar cells promotes infiltration of myeloid cells (CD11b^+^), including MDSCs, macrophages, and M2 macrophages, and decreases infiltration of CD3^+^CD8^+^ and CD3^+^CD4^+^ T cells and T cell activation in the pancreata of KC mice. Our data suggested that loss of HPK1 kinase activity in pancreatic epithelial cells promotes an immunosuppressive microenvironment, which may in turn promote the progression of *Kras^G12D^*-driven PanIN formation and progression to PDAC. While the results from ongoing clinical trials that target HPK1 kinase activity in immune cells for cancer immunotherapy remain to be determined, our results demonstrated that targeting of HPK1 kinase activity is a double-edged sword for patients either harboring mutant *Kras* or at increased risk for pancreatic cancer. Although inhibition of HPK1 kinase activity has been shown to enhance T cell activation and may potentially improve tumor response to immunotherapies for patients with solid tumors, it may also promote the activation of Ras and its downstream signaling and an immunosuppressive microenvironment as well as enhance the tumor development mediated by oncogenic mutant *Kras*. *Kras* mutations have been reported to be present in the pancreata of approximately 25% of the normal population and in patients with chronic pancreatitis (56–58)**.** Our study provides a better understanding of how HPK1 impacts *Kras*-induced pancreatic tumorigenesis and tumor microenvironment, which is critically needed for future development of immunotherapeutic strategies targeting HPK1.

Defining tumor-initiating events and the molecular mechanisms that lead to the progression of pancreatic precursor lesions to invasive PDAC is critically important for the development of early-detection methods and effective treatments. Currently, many fundamental questions remain to be answered regarding pancreatic cancer initiation and progression. How do acinar cells sense the genetic and environmental stress? What are the key factors that drive acinar-to-ductal cell reprogramming? How do acinar cells and/or ductal cells maintain their cellular identity? HPK1 appears to be a gatekeeper for the progression of early precursor lesions, such as ADM and PanIN1, to high-grade PanINs and PDAC. Future studies using HPK1-knockout mice may further define the functions of HPK1 in the feed-forward loop among oncogenic mutant *Kras*, inflammation, and the tumor microenvironment to promote pancreatic cancer progression.

## Methods

### Generation of conditional HPK1- or M46-transgenic mice.

Full-length cDNAs encoding HPK1 or its kinase-dead mutant, M46, were subcloned into the HindIII and NotI sites in pCAGGS vector behind a *loxP*-GFP-stop-*loxP* (LSL) site. The CMV-LSL-HPK1 (hereafter referred to as HPK1) cassette and the CMV-LSL-M46 (hereafter referred to as M46) cassette were isolated by PmeI digestion and submitted for pronuclear injection. The GFP expression in pups of transgenic mice was visualized under a UV lamp.

### Mouse breeding and treatment.

To target the expression of transgenes in pancreatic acinar cells, *HPK1*- or *M46*-transgenic mice were crossed with tamoxifen-regulated *Bac-cre* mice, in which Cre was driven by a full-length pancreatic acinar-specific elastase I promoter, and transgene expression was induced with daily tamoxifen treatment (oral gavage, 3 mg/40 g body weight for 5 days). *LSL-Kras^G12D^* mice were obtained from the Mouse Models for Human Cancer Consortium Repository (Rockville, Maryland, USA). These mouse strains were interbred to generate experimental cohorts with the following genotypes: *HPK1 Bac-cre* (HC), *M46 Bac-cre* (MC), *LSL-Kras^G12D^ HPK1 Bac-cre* (KHC), *LSL-Kras^G12D^ M46 Bac-cre* (KMC), and *LSL-Kras^G12D^ Bac-cre* (KC). Mouse strains were genotyped by PCR as previously described (25, 30). Mice were either treated with 8 hourly intraperitoneal injections of cerulein (50 μg/kg/h; ICer) per day for 2 days to induce acute pancreatitis or treated with 6 hourly injections per day 3 days per week for 2 weeks to induce chronic pancreatitis. Littermate control mice received PBS injections. Mice were euthanized at different time points after the treatment.

### RNA-Seq for transcriptome analyses.

Total RNA was isolated from the mouse pancreatic tissue or mouse PDAC samples using the RNeasy Mini/Micro Kit (Qiagen) according to the manufacturer’s instructions. RNA quality was measured by an Agilent Bioanalyzer. The total RNA samples with RNA integrity numbers ≥7 were processed for RNA-Seq transcriptome profile analysis. RNA-Seq was performed at the Advanced Technology Genomics Core of The University of Texas MD Anderson Cancer Center with NovaSeq, generating 100-bp paired-end reads. The sample library was prepared using Illumina TruSeq stranded protocol. RNA-Seq FASTQ files were processed through FastQC (https://www.illumina.com/products/by-type/informatics-products/basespace-sequence-hub/apps/fastqc.html), a quality control tool to evaluate the quality of sequencing reads at both the base and read levels. Samples that passed quality control were taken into subsequent analysis. STAR alignment to the Genome Reference Consortium Mouse Build 38 (GRCm38) reference geome was performed with default parameters to generate RNA-Seq BAM files. Aligned reads were summarized at the gene level using STAR (59). Gene-level annotation was carried out using the GENCODE annotation, which was downloaded from the GENCODE project (60). The raw count data were processed and normalized by DESeq2 software to identify differentially expressed genes (DEGs) between 2 groups (61). The final *P* value was adjusted using the Benjamini-Hochberg method (62). A cutoff of gene expression log_2_ fold change of ≥1.0 or ≤ –1.0 and an FDR *q* value of ≤0.05 were applied to select the most significant DEGs. Differential expression analysis was further evaluated using the pathway enrichment tool GSEA (63). Comparative bioinformatics statistical analyses for the RNA-Seq raw data were performed by 2 experts at the Department of Bioinformatics & Computational Biology at MD Anderson Cancer Center. A gene set enrichment assay for the RNA-Seq results was analyzed using the R4.1.0 package ClusterProfiler and other associated R packages. The full RNA-Seq data were deposited in the NCBI’s public Gene Expression Omnibus database (GEO GSE223215).

### Histopathologic and immunohistochemical analysis.

Mouse pancreata were fixed in 10% formalin and embedded in paraffin. Immunohistochemical (IHC) staining for HPK1, p-Erk, p-HPK1^S171^, Ki-67, CK19, F4/80, and CD206 was performed at 4°C overnight. Alcian blue and sirius red stains were performed using commercial kits. Histopathologic and IHC analyses were performed independently by 2 pathologists.

### Antibody array-based screening for HPK1-interacting proteins in pancreatic cancer cells.

The cell lysates from the Panc-1/*HPK1* stable cells treated with 2.0 μM MG132 for 16 hours, which stabilized HPK1 protein, were incubated with a membrane filter arrayed with 400 antibodies (Hypromatrix) overnight at 4°C, then detected by horseradish peroxidase–conjugated anti-FLAG antibody, followed by chemiluminescence according to the manufacturer’s protocol.

### Immunoprecipitation and immunoblotting.

Immunoprecipitation and immunoblotting were performed as described previously (28). For each coimmunoprecipitation, 500 μg of total proteins were mixed with 2 μg of antibody or normal IgG. The precipitated proteins were separated by 10% SDS-PAGE, then electroblotted onto PVDF membranes (Novex), blocked in 5% skim milk in 1× TBST, and probed with primary antibodies indicated in the figure legends.

### Measurement of GTP- and GDP-bound Ras using thin-layer chromatography.

The HEK293T cells were cotransfected with HA-Kras and FLAG-HPK1 or FLAG-M46. The transfected cells were serum-starved overnight, treated with EGF, switched to phosphate-free medium for 30 minutes, and then incubated with phosphate-free medium containing 150 μCi ^32^Pi per 60-mm dish for 4 hours. After labeling, the cells were washed with ice-cold PBS. The cell lysates were centrifuged at 16,000*g* at 4°C for 10 minutes. The supernatant was used for immunoprecipitation using HA beads at 4°C for 1 hour. Nucleotides bound to GTPase were released by denaturing of the protein in 20 μL of elution buffer at 65°C for 5 minutes. The nucleotides were separated by polyethylenimine cellulose plates saturated with 0.75 M KH_2_PO_4_ (pH 3.4). The plates were air-dried, visualized by x-ray film, and ImageJ software (https://imagej.nih.gov/ij/download.html).

### Ras activity assay.

Cells or pancreata were lysed on ice in 25 mmol/L HEPES (pH 7.5). Equal amounts of protein were incubated for 45 minutes at 4°C with beads coated with Raf1-RBD, then washed 3 times with ice-cold lysis buffer. The bound protein was eluted for 15 minutes with 2× Laemmli sample buffer that had been preheated to 95°C and analyzed by immunoblotting.

### Primary pancreatic acinar cell culture.

The pancreata from the HC mice and control littermates that had been treated with tamoxifen were digested with digestion buffer at 37°C for 30 minutes as described previously (64). The acinar cells were isolated, equally seeded into a type I collagen–coated 6-well dish, and cultured at 37°C overnight. The cells were treated with cerulein for 30 minutes, 1 hour, and 2 hours as indicated.

### Immunocomplex kinase assays.

HPK1 or M46 was immunoprecipitated using the anti-FLAG antibody, and the immunocomplex kinase assays were performed as described previously (9). The kinase reaction was performed at 30°C for 30 minutes and terminated with an equal volume of SDS sampling buffer. The reaction mixtures were resolved by SDS-PAGE analysis.

### Immune cell profiling by flow cytometry.

Mouse pancreata were digested in digestion buffer (1 mg/mL collagenase IV and 10 U/mL DNase I in DMEM) at 37°C for 30 minutes, pushed through a 70-μm cell strainer, and washed by 40 mL DMEM without FBS. The digested cells were resuspended in 37% Percoll and overlaid on top of the same volume of 70% Percoll. After centrifugation at 800*g* for 20 minutes with the brake off, the lymphocytes were isolated from the interface and rinsed with PBS. The cells were first stained by Zombie UV (BioLegend), then were incubated with a cocktail comprising the following antibodies in cell staining buffer: anti-CD45, anti-CD3ε, anti-B220, anti-CD11b, anti-F4/80, anti-Ly6C, anti-Ly6G, anti-CD4, anti–IFN-γ, anti-IA/IE, and anti-CD8α. After washing, the stained cells were submitted for multiple-color flow cytometry. The results were processed using FlowJo software (BD Biosciences).

All antibodies, chemicals, peptides, recombinant proteins and cell lines used are listed in Supplemental Table 1.

### Statistics.

Statistical significance was determined by unpaired Student’s *t* test or ANOVA for comparison of the means among groups or by χ^2^ analysis for comparison of the frequencies among groups. Survival curves were constructed using the Kaplan-Meier method, and the log-rank test was used to determine the significance of survival differences. All tests were 2-sided, and significance was defined as *P* less than 0.05. Data were analyzed using SPSS software v26 or GraphPad Prism v7.01.

### Study approval.

All animal experiments were conducted under a protocol approved by the Institutional Animal Care and Use Committee at The University of Texas MD Anderson Cancer Center.

## Author contributions

Hua Wang designed and performed experiments, bred and genotyped mice, collected and analyzed data, and drafted and revised the manuscript. RM designed and performed experiments, bred and genotyped mice, collected and analyzed data, and revised the manuscript. LL designed experiments, bred and genotyped mice, and collected and analyzed data. BJ performed transgenic mouse experiments, analyzed data, and revised the manuscript. HT performed RNA-Seq and analyzed data. RA performed experiments and collected and analyzed data. JZ performed histopathologic and immunohistochemical analysis of mouse pancreata. GL performed immunoprecipitation and collected and analyzed data. RW processed and analyzed RNA-Seq data. XS performed RNA-Seq and analyzed data. YL analyzed flow cytometric and RNA-Seq data. XZ analyzed flow cytometric data and revised the manuscript. THT performed study design and analyzed data for HPK1-knockout mice, and revised the manuscript. AM performed study design, analyzed and interpreted data, and revised the manuscript. RS analyzed RNA-Seq data, performed statistical analysis, and revised the manuscript. Huamin Wang performed overall study design, funding acquisition, and supervision, analyzed and interpreted data, and drafted and revised the manuscript.

## Supplementary Material

Supplemental data

## Version 1 05/04/2023

In-Press Preview

## Version 2 06/15/2023

Electronic publication
