# Supplementary material for: Hematopoietic progenitor kinase 1 inhibits the development and progression of pancreatic intraepithelial neoplasia
Source: J Clin Invest. 2023 Jun 15;133(12):e163873. doi: 10.1172/JCI163873 (PMC10266776; doi:10.1172/JCI163873)
Supplement: Supplemental data [file jci-133-163873-s160.pdf]

Hematopoietic Progenitor Kinase 1 Inhibits the Development and Progression of Pancreatic Intraepithelial Neoplasia

SUPPLEMENTAL DATA

Supplemental Table 1. Materials and Reagents

Antibodies	Sources	Identifiers
Rabbit HPK1 antibody	Cell Signaling Technology	4472
Rabbit phospho-ERK1/2	Cell Signaling Technology	4370S
Mouse RasGAP antibody	Santa Cruz Biotechnology	SC-63
Goat HPK1 antibody (IHC)	Santa Cruz Biotechnology	SC-6231
Rabbit anti-CK19	Abcam	AB52625
P-HPK1^{ser171} antibody	Invitrogen	pA-5-12881
Rabbit ERK1/2	Cell Signaling Technology	9102
PE monoclonal anti-mouse I-A/I-E (F)	BioLegend	107607
PerCP/Cy5.5 monoclonal anti-mouse CD45 (F)	BioLegend	103131
Rat Brilliant Violet 785 monoclonal anti-mouse CD3ε (F)	BioLegend	100355
Rat Alexa Fluor 700 monoclonal anti-mouse/human CD45R/B220 (F)	BioLegend	103231
Rat Brilliant Violet 510 monoclonal anti-mouse Ly-6G/Ly-6C (Gr1) (F)	BioLegend	108438
Rat PE/Cy7 monoclonal anti-mouse/ human CD11b (F)	BioLegend	101215
Rat Brilliant Violet 421 monoclonal anti-mouse F4/80 (F)	BioLegend	123131
Rat Brilliant Violet 510 monoclonal anti-mouse Ly6C (F)	BioLegend	128033
Rat APC/Fire 750 monoclonal anti-mouse Ly6G (F)	BioLegend	127651
Rat BUV737 monoclonal anti-mouse CD8α (F)	BD Biosciences	564297
Rat FITC monoclonal anti-mouse CD4 (F)	BioLegend	100405
TruStain FcX (anti-mouse CD16/32) antibody (F)	BioLegend	101319
Biotinylated goat anti-rabbit IgG antibody	Vector Laboratories	BA-1000
Goat anti-rat IgG (H+L) secondary antibody, Alexa Fluor 594 conjugated	Thermo Fisher Scientific	A-11007
Goat anti-rabbit IgG (H+L) secondary antibody, Alexa Fluor 488 conjugated	Thermo Fisher Scientific	A-11034
Goat anti-rabbit IgG (H+L) secondary antibody, Alexa Fluor 594 conjugated	Thermo Fisher Scientific	A-11012
M2 (Anti-Flag)	Sigma	F3165
Anti-Ras antibody	Millipore	05-516
Rabbit Ki-67	Abcam	AB16667
Anti-Phosphoserine/threonine antibody	invitrogen	PA5-121314
Anti-actin antibody	Santa Cruz	SC-1616

Anti-HA antibody	Santa Cruz	SC-805
Anti-HSP90 antibody	Santa Cruz	SC-7947
F4/80 antibody (IHC)	Cell signaling	70076S
CD206 antibody (IHC)	Cell signaling	#24595S
**Chemicals, Peptides, and Recombinant Proteins**	**Sources**	**Identifiers**
RBC lysis buffer (10X)	BioLegend	420301
Cell staining buffer	BioLegend	420201
NovaUltra Alcian blue stain kit	IHCWORLD	IW-3000
NovaUltra Sirius red stain kit	IHCWORLD	IW-3012
Zombie UV Fixable Viability Kit	BioLegend	423107
ArC Amine Reactive Compensation Bead kit	Thermo Fisher Scientific	A10628
UltraComp Beads for Ab single stains	Thermo Fisher Scientific	01-2222-41
Precision Count Beads	BioLegend	424902
Ras Activation Assay Kit	Millipore	17-218
Myelin basic protein (MBP)	Abcam	Ab43614
EGF	Sigma-Aldrich	SRP3027
Ras GAP (277-346) AC<br>Ras GAP (343-442) AC<br>Ras GAP (175-274) AC<br>Ras GAP (171-448) AC	Santa Cruz<br>Santa Cruz<br>Santa Cruz<br>Santa Cruz	SC-4056 AC<br>SC-4057 AC<br>SC-4055 AC<br>SC-4018 AC
Antibody-based Array	Hypromatrix	HM3000
**Cell lines**	**Sources**	**Identifiers**
Panc1 cells	ATCC	CRL-1469
Panc-1/HPK1 stable cells	Dr. Huamin Wang's Lab	Wang H et al. Cancer Research 2009;69(3):1063-70
HEK293T cells	ATCC	CRL-3216
**Experimental Mouse Models**	**Sources**	**Identifiers**
Mouse: C57BL/6J (B6)	Jackson Laboratory	664
Mouse: LSL-KRas^{G12D} (B6.129S4-*Kras*^{tm4Tyj}/J)	Jackson Laboratory	008179
Mouse: Ela-CreERT (Bac-cre)	Generated by Dr. Baoan Ji	N/A
Mouse: Pdx-cre	Jackson Laboratory	014647
Mouse: CAG-LSL-HPK1-Flag	Generated by our lab	N/A
Mouse: CAG-LSL-M46-Flag	Generated by our lab	N/A
Mouse: HPK1 knockout mice	~~Dr. Tse-Hua Tan Lab~~	~~Shui *et al.* Nature Immunology 2007, 8(1):84-91~~

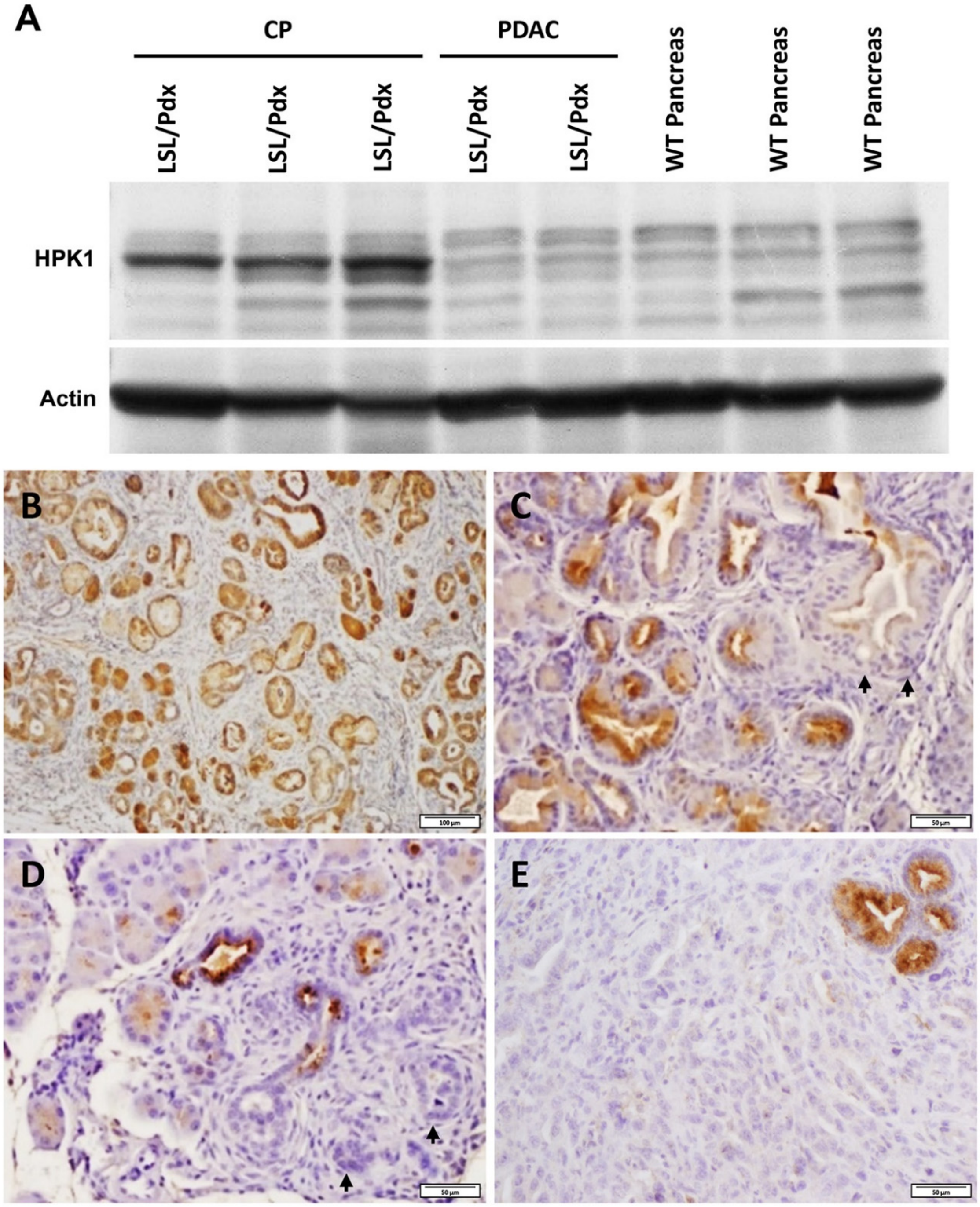

Supplemental Figure 1. HPK1 is expressed in normal pancreatic ducts, chronic pancreatitis, low-grade PanINs, but lost in high-grade PanINs and invasive pancreatic ductal adenocarcinoma (PDAC) from *LSL-KRas*^{G12D}*;Pdx1-cre* mice. HPK1 is expressed in the normal pancreatic ductal cells in chronic pancreatitis and PanIN1 (A and B), but lost in PanIN2 (B, marked by arrows), PanIN3 (C, marked by arrows) and PDAC (D). The positive staining for HPK1 in normal pancreatic ductal cells serves as internal positive control for the HPK1 immunohistochemistry in B-E.

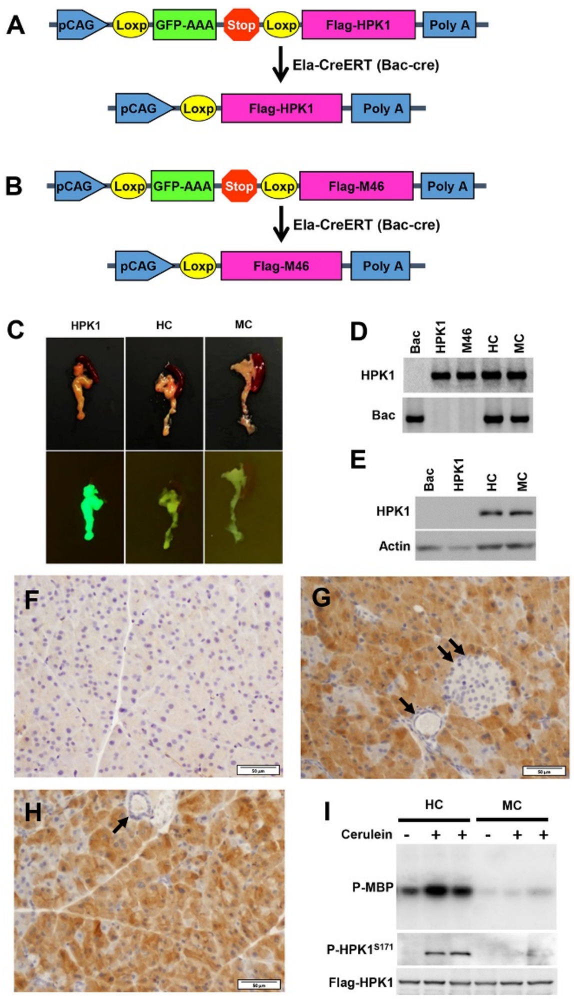

Supplemental Figure 2. Generation of transgenic mouse models for pancreatic acinar cell-specific expression of *HPK1* or its kinase-dead mutant, *M46*. A and B. We engineered *HPK1* or *M46* cDNA downstream of a human CMV and chicken-β-actin chimeric promoter and *HPK1* or *M46* expression is blocked by the proximal insertion of a lox-green fluorescent protein (*GFP*)-stop-lox cassette in pCAGGS vector. Schematic drawings of the *HPK1* or *M46* transgene structures before and after pancreatic specific cre-mediated recombination are shown. These constructs have been microinjected into pronuclei by the Transgenic Core at MD Anderson Cancer Center. The pups harboring *HPK1* or *M46* transgenes showed GFP fluorescence in whole body. C. Loss of green fluorescence in mouse pancreata of HC and MC mice compared to *HPK1* mice, demonstrating the efficacy of *Bac-cre*. D. Mouse genotyping PCR results. E. Immunoblotting shows HPK1 expression in the pancreata of HC and MC mice compared to the controls. F-H. Immunohistochemical stains show the expression of HPK1 in wild type (F), HC (G) and MC mice (H). As expected, immunohistochemical stains show that HPK1 or M46 was expressed only in pancreatic acinar cells, but not in the ductal cells (single arrow) and islet cells (double arrows) in HC or MC mice. I. The pancreata of HC mice have high basal and caerulein-induced HPK1 kinase activities and high expression of P-HPK1^{S171} while no or minimal kinase activity was detected in the pancreata of both untreated or treated MC mice.

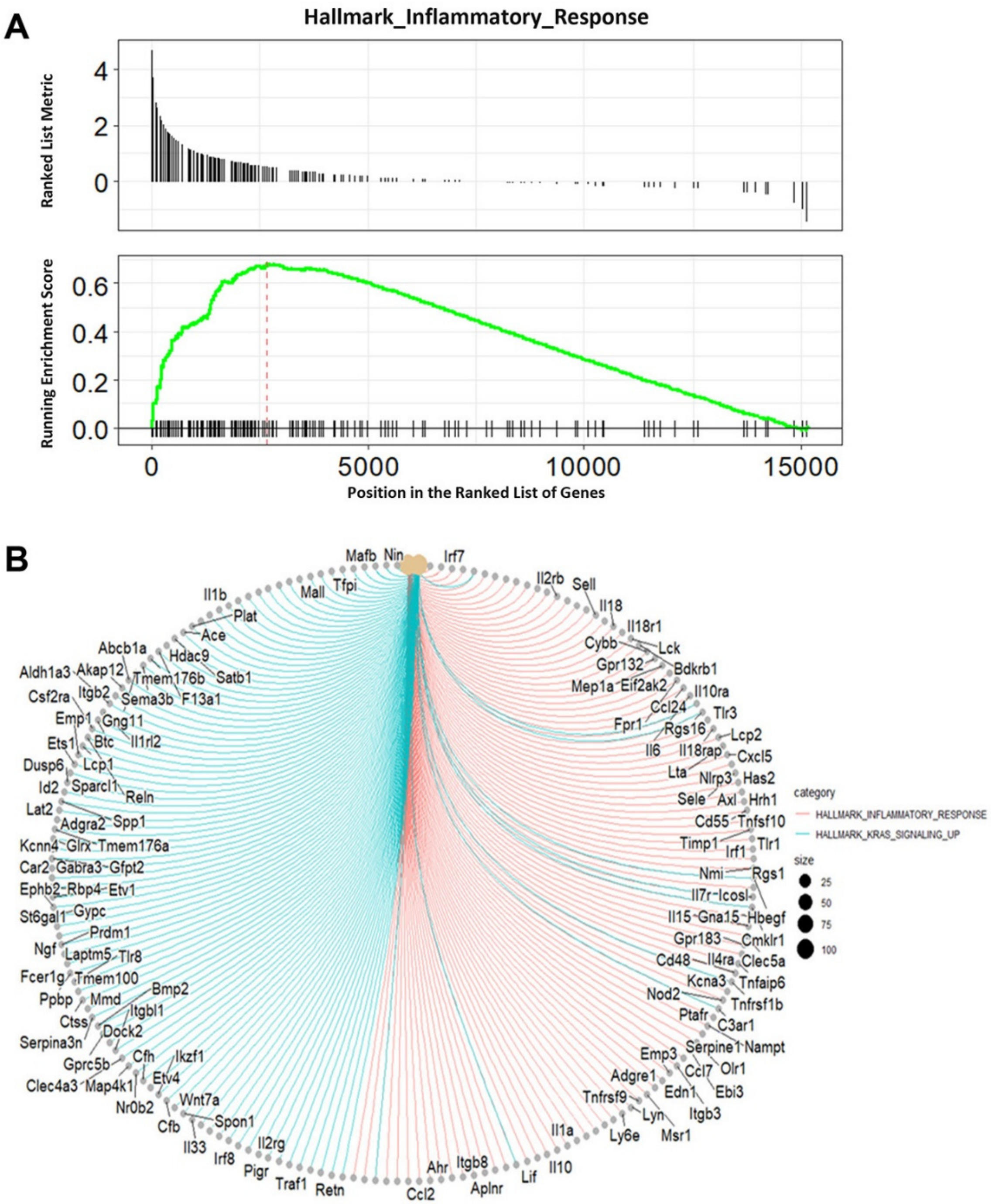

Supplemental Figure 3. A. Expression of M46 is associates with upregulation of hallmark inflammatory response gene set in MC mice. Six to eight weeks old MC and *Bac-cre* mice (n=6 for each group) were treated with tamoxifen (TM) for 5 days then treated with 8 doses of intra-peritoneal injection of cerulein to induce acute pancreatitis. The pancreata were harvested 24 hours after ICer. A. GSEA plots of the enriched inflammation-related pathways. B. Cnetplot of Gene Set Enrichment Analysis demonstrates the network of differentially expressed genes in the Kras signaling up and inflammatory response pathways in pancreatic acinar cells of MC mice.

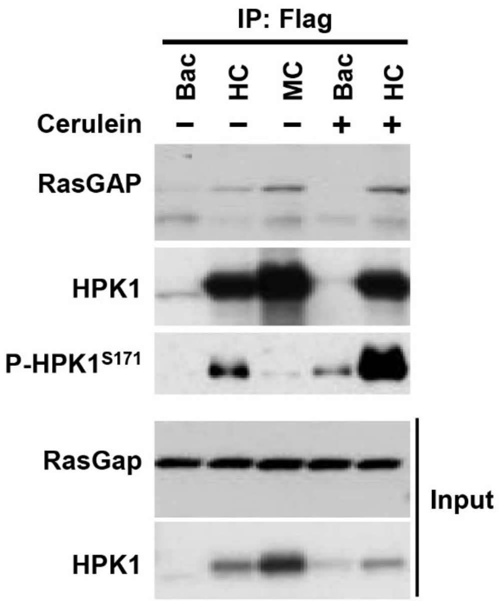

Supplemental Figure 4. HPK1 interacted with endogenous RasGAP in mouse pancreata. The Bac control, HC and MC mice were either untreated or treated with one dose of intraperitoneal cerulein injection to activate HPK1 and Ras pathways and their pancreata were harvest at 1 hour after the treatment. The interaction between HPK1 or M46 with RasGAP in the pancreata of HC and MC mice was detected by coimmunoprecipitation. No difference in HPK1 binding to RasGAP was observed between the treated and untreated HC mice. Immunoblotting for P-HPK1^{S171} showed HPK1 autophosphorylation in the pancreata of HC mice, but not in the pancreata of MC mice.

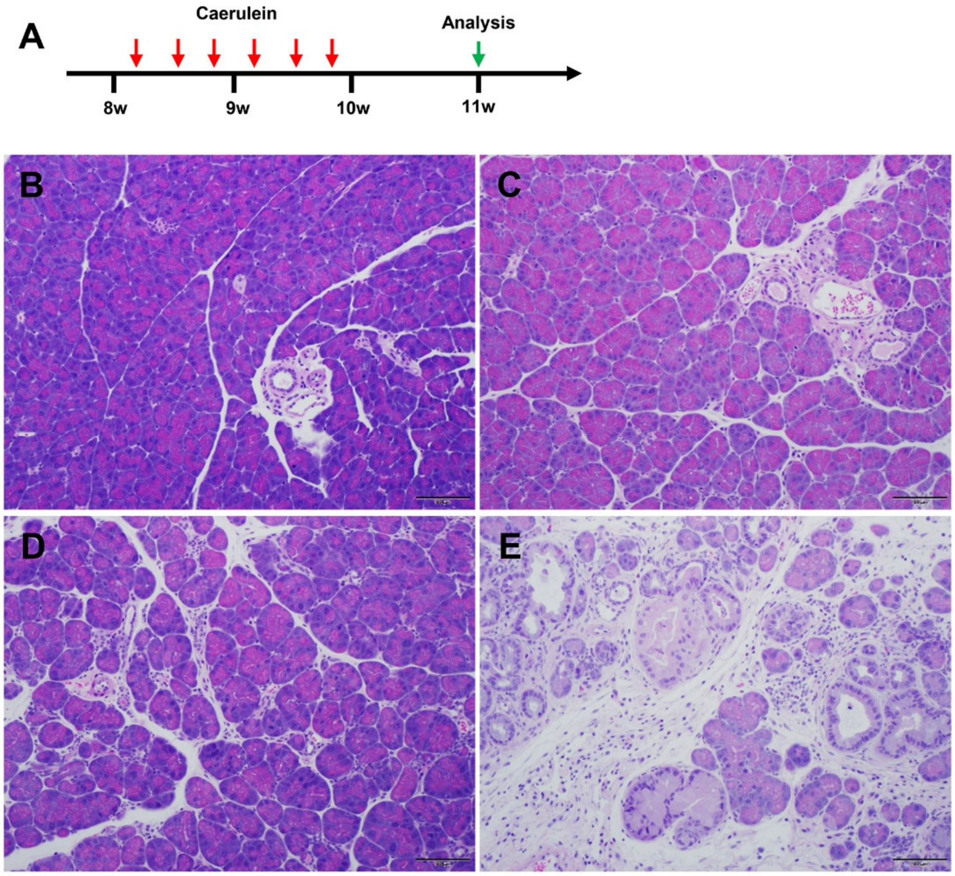

Supplemental Figure 5. Pancreatic inflammation, ADM and PanIN formation in HPK1 knockout mice. A. Scheme of cerulein treatment and tissue harvesting. B and C, Representative micrographs of the pancreata (H & E stain) from untreated wild control (B) and HPK1 knockout mice (C). The pancreas in untreated HPK1 knockout mice is grossly and histologically normal. D and E, Representative micrographs of the pancreata (H & E stain) from wild control and HPK1 knockout mice after treated with ICer. Multifocal PanIN formation and ADM were observed in the pancreata of HPK1 knockout mice after ICer treatment (E), which were not present in treated wild type control mice (D).

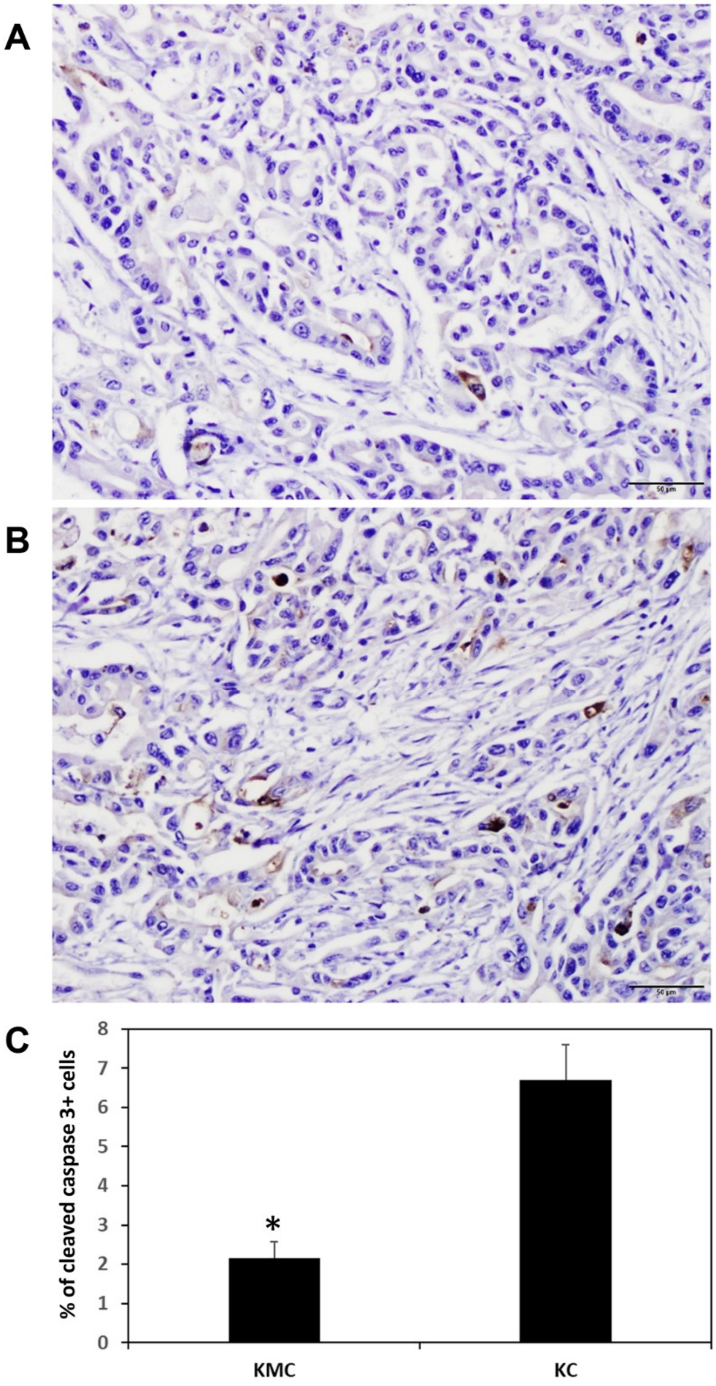

Supplemental Figure 6. Representative micrographs showing the expression of cleaved caspase 3 in PDACs from KMC mice (A) compared to those from KC mice (B). C. PDACs from KMC mice have lower number of cleaved caspase 3+ cells than those from KC mice. Data are presented as mean ± SEM. Unpaired t-test was used to determine the statistical significance. Statistically significant difference was marked by *.

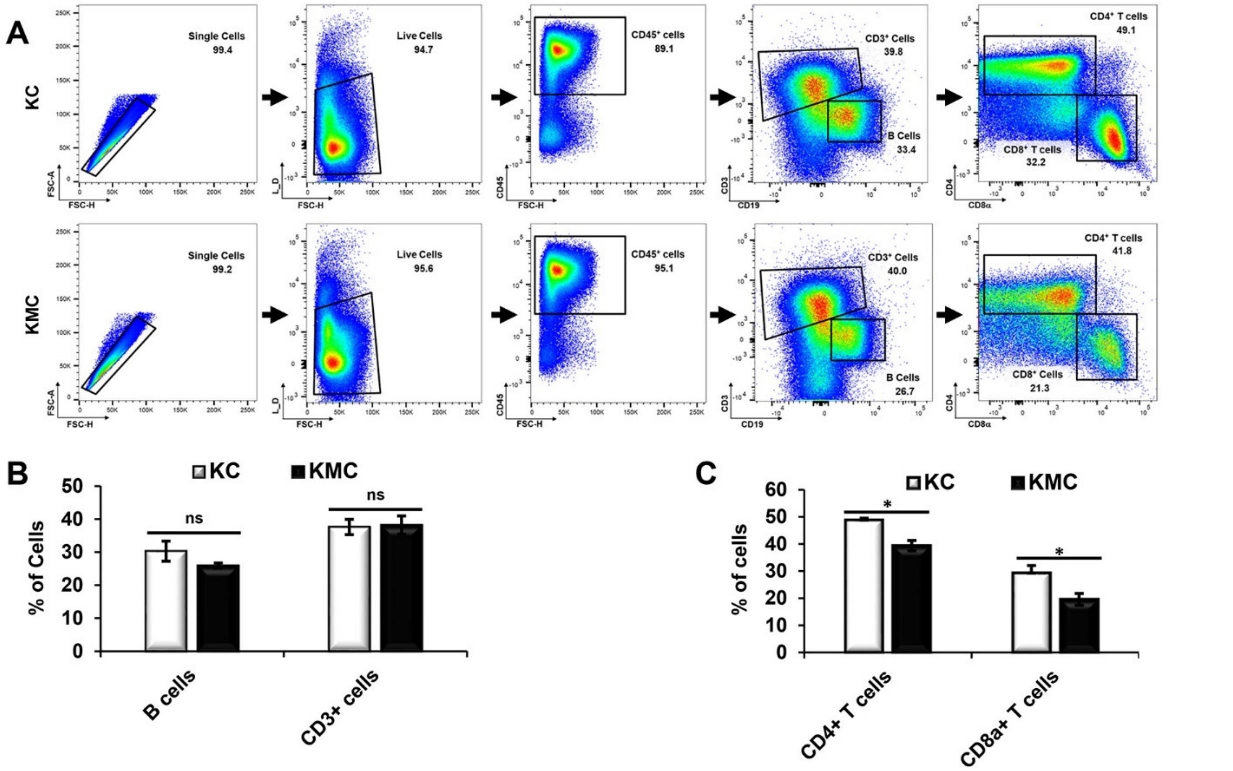

Supplemental Figure 7. Kinase-dead mutant M46 inhibits the infiltration of CD4$^+$ and CD8 $^+$ T cells in the pancreata of KC mice. KC and KMC mice at 6 months were euthanized (n = 6 per group). The infiltrating lymphocytes in pancreatic tissues were quantified by flow cytometry. A. Representative flow cytometry gating results for infiltrating CD45$^+$ cells, including B cells (CD19$^+$), T cells (CD3$^+$) cells, CD3$^+$CD4$^+$ cells, and CD3$^+$CD8$^+$T cells. B. Quantitative results for B cells and T cells. C. CD3$^+$CD4$^+$ cells and CD3$^+$CD8$^+$T cells. Data are presented as mean ± SEM. Unpaired t-test was used to calculate p values. *P<0.05; **p<0.01; ns, not significant.

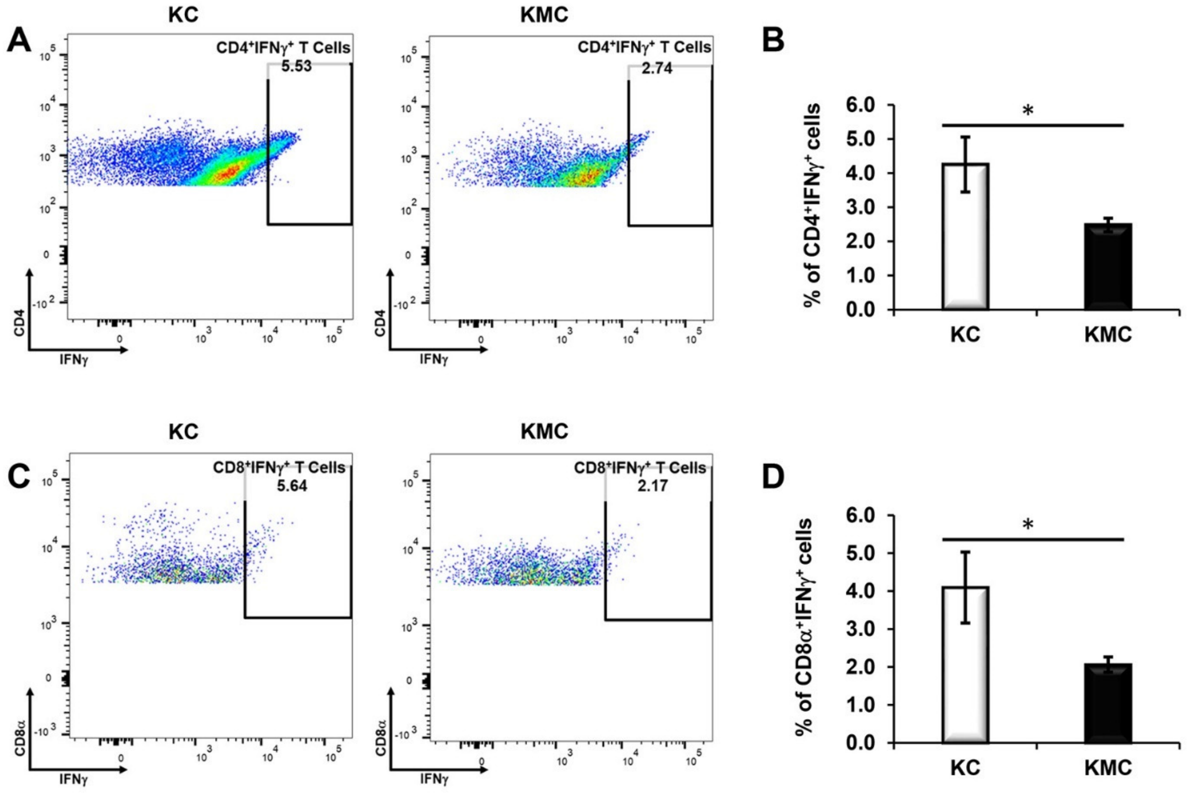

Supplemental Figure 8. Expression of kinase-dead mutant M46 inhibits the activation of CD4^{+} and CD8^{+} T cells in the pancreata of KC mice. KC and KMC mice at 6 months were euthanized (n = 6 per group). The infiltrating CD4^{+} IFNγ^{+} and CD8^{+} IFNγ^{+} lymphocytes in pancreatic tissues were quantified by flow cytometry. A. Representative flow cytometry plots of CD4^{+} T cells, gating on IFNγ-producing cells. B. Quantitative results for CD4^{+} IFNγ^{+} lymphocytes. C. Representative flow cytometry plots of CD8^{+} T cells, gating on IFNγ-producing cells. B. Quantitative results for CD8^{+} IFNγ^{+} Lymphocytes. B and D. Data are presented as mean ± SEM. Unpaired t-test was used to calculate p values (*P<0.05).

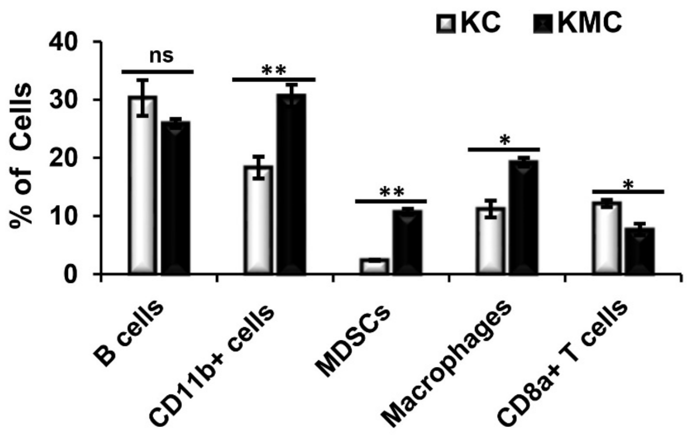

Supplement Figure 9. Kinase-dead mutant M46 enhances proinflammatory immune response in KC mice. The KC and KMC mice at 6 months were euthanized (n = 6 per group). The infiltrating immune cells in pancreatic tissues were quantified by flow cytometry. Quantitative results of pancreas-infiltrating $CD45^+$ cells, including B cells, CD11b cells ($CD11b^+$), total myeloid-derived suppressor cells (MDSCs), macrophages ($CD11b^+F4/80^+$), and $CD3^+CD8^+$T cells. Data are presented as mean ± SEM. Unpaired t-test was used to calculate p values. *P<0.05; **p<0.01; ns, not significant.
